# Sympatry or syntopy? Investigating drivers of distribution and co‐occurrence for two imperiled sea turtle species in Gulf of Mexico neritic waters

**DOI:** 10.1002/ece3.4691

**Published:** 2018-11-26

**Authors:** Kristen M. Hart, Autumn R. Iverson, Ikuko Fujisaki, Margaret M. Lamont, David Bucklin, Donna J. Shaver

**Affiliations:** ^1^ Wetland and Aquatic Research Center U.S. Geological Survey Davie Florida; ^2^ Wetland and Aquatic Research Center CNT, Contracted to U.S. Geological Survey Davie Florida; ^3^ Ft. Lauderdale Research and Education Center University of Florida Davie Florida; ^4^ Wetland and Aquatic Research Center U.S. Geological Survey Gainesville Florida; ^5^ Padre Island National Seashore National Park Service Corpus Christi Texas

**Keywords:** *Caretta caretta*, foraging, Gulf of Mexico, *Lepidochelys kempii*, principal component analysis, satellite tracking, sea turtle, switching state‐space modeling

## Abstract

Animals co‐occurring in a region (sympatry) may use the same habitat (syntopy) within that region. A central aim in ecology is determining what factors drive species distributions (i.e., abiotic conditions, dispersal limitations, and/or biotic interactions). Assessing the degree of biotic interactions can be difficult for species with wide ranges at sea. This study investigated the spatial ecology of two sea turtle species that forage on benthic invertebrates in neritic GoM waters: Kemp's ridleys (*Lepidochelys kempii*) and loggerheads (*Caretta caretta*). We used satellite tracking and modeled behavioral modes, then calculated individual home ranges, compared foraging areas, and determined extent of co‐occurrence. Using six environmental variables and principal component analysis, we assessed similarity of chosen foraging sites. We predicted foraging location (eco‐region) based on species, nesting site, and turtle size. For 127 turtles (64 Kemp's ridleys, 63 loggerheads) tracked from 1989 to 2013, foraging home ranges were nine to ten times larger for Kemp's ridleys than for loggerheads. Species intersected off all U.S. coasts and the Yucatán Peninsula, but co‐occurrence areas were small compared to species' distributions. Kemp's ridley foraging home ranges were concentrated in the northern GoM, whereas those for loggerheads were concentrated in the eastern GoM. The two species were different in all habitat variables compared (latitude, longitude, distance to shore, net primary production, mean sea surface temperature, and bathymetry). Nesting site was the single dominant variable that dictated foraging ecoregion. Although Kemp's ridleys and loggerheads may compete for resources, the separation in foraging areas, significant differences in environmental conditions, and importance of nesting location on ecoregion selection (i.e., dispersal ability) indicate that adult females of these species do not interact greatly during foraging and that dispersal and environmental factors more strongly determine their distributions. These species show sympatry in this region but evidence for syntopy was rare.

## 1 INTRODUCTION

Understanding how factors such as abiotic conditions, dispersal limitations, and biotic interactions (Soberón, 2007) influence species distributions is a central aim in the field of ecology. Abiotic conditions are often used as indicators of habitat suitability and may set the “fundamental” or “Grinnellian” niche (Hutchinson, 1957; Soberón, 2007), be used in species distribution modeling (Austin, 2002), and predict future impacts of environmental changes (Franklin, 2010). However, it has long been recognized that environment alone cannot fully explain species distributions and that biotic interactions (e.g., predatory/prey relationships, competition) determine the “realized” or “Eltonian” niche within the wider environment‐defined niche (Hutchinson, 1957; Soberón, 2007). Species distribution models are beginning to be improved by incorporating biotic interactions (Kissling et al., 2011; Pollock et al., 2014) but assessing the degree of sympatric biotic interactions at a site can be more difficult than classifying environmental variables, especially for species with wide ranges at sea.

Five sea turtle species occur in the Gulf of Mexico (GoM): green turtles (*Chelonia mydas*; Hart, Zawada, Fujisaki, & Lidz, 2013), hawksbills (*Eretmochelys imbricata;* Hart, Sartain, et al., 2012), Kemp's ridleys (*Lepidochelys kempii*, Shaver et al., 2013), leatherbacks (*Dermochelys coriacea*; Epperly et al., 2002; Fritts, Hoffman, & McGehee, 1983), and loggerheads (*Caretta caretta*, Hart, Lamont, Sartain, & Fujisaki, 2014). Green turtles primarily eat seagrass (Thayer, Bjorndal, Ogden, Williams, & Zieman, 1984), hawksbills generally inhabit coral reefs and are considered spongivores (Meylan, 1988; Mortimer & Donnelly, 2008), and leatherbacks specialize on jellyfish (Houghton, Doyle, Wilson, Davenport, & Hays, 2006), making these species unlikely to compete or have similar biotic (i.e., prey) drivers to their distribution. Loggerheads and Kemp's ridleys, however, both forage primarily in shallow waters on benthic invertebrates (Bjorndal, 1997; Shaver, 1991), making competition or a syntopic overlap between these two species plausible. Additionally, Kemp's ridleys and loggerheads are found throughout GoM neritic waters, and both are found in the GoM during every stage of their lives (Lamont, Putman, Fujisaki, & Hart, 2015). They nest on sandy GoM beaches: Kemp's ridleys nest from approximately April to July primarily on the coasts of Mexico and Texas but also sporadically in other Gulf states including Alabama and Florida, and loggerheads nest from April to September along the entire GoM coast from Mexico to the Dry Tortugas, Florida.

Here, we use satellite tracking data for Kemp's ridley and loggerhead sea turtles to assess how they distribute themselves across GoM neritic waters and what drivers may be important to their distribution. We delineate home ranges, quantifying the degree of species spatial and temporal co‐occurrence. To assess abiotic drivers, we characterize habitat variables and foraging ecoregions and compare them across species. Together, these tools help us understand and compare how these species utilize nearshore GoM waters, what factors influence this distribution, and whether these two imperiled species show evidence for sympatry or syntopy in this region.

## 2 MATERIALS AND METHODS

### 2.1 Turtle capture and tracking

**Figure 1:**
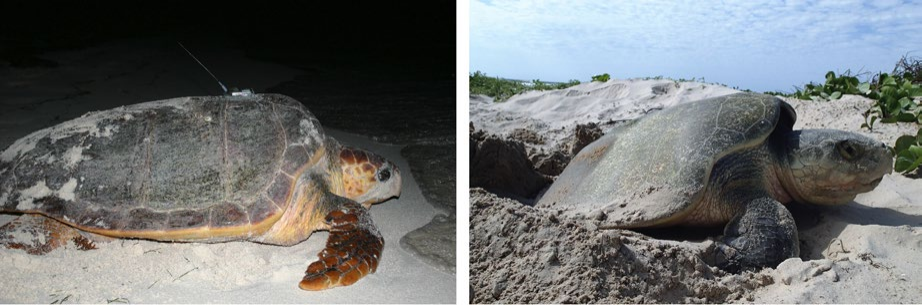
Satellite‐tagged loggerhead (*Caretta caretta*) female heading back to the water after nesting (left panel) and a female Kemp's ridley (*Lepidochelys kempii*) during nesting (right panel). Images taken with permission (MTP 176 issued to KH and USFWS permit TE840727‐3 issued to DS) under conditions not harmful to turtles

**Figure 2:**
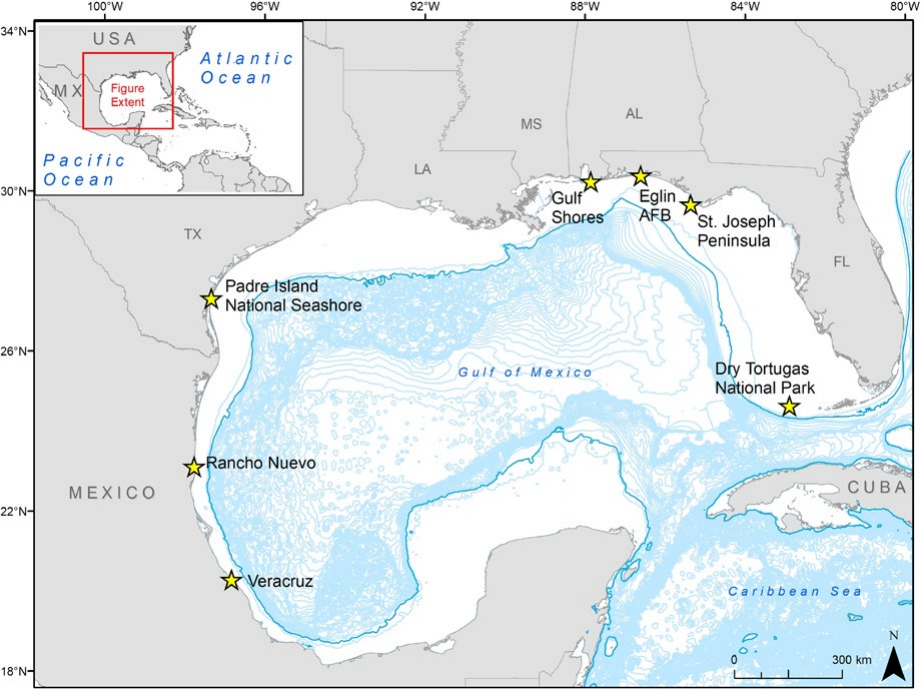
Tagging locations in the Gulf of Mexico (yellow stars) for Kemp's ridley (*Lepidochelys kempii*) and loggerhead (*Caretta caretta*) sea turtles. Blue lines represent 100 m bathymetry contour intervals; darker blue line is −200 m

We tagged Kemp's ridley and loggerhead females (Figure 1) with satellite transmitters after they nested on beaches along the GoM (Table 1, Figure 2). Tagging, including attaching platform terminal transmitters (PTTs), followed our previous studies (Hart et al., 2015; Shaver et al., 2016) and established protocols (National Marine Fisheries Service [NMFS] Southeast Fisheries Science Center, 2008). Models included Wildlife Computers (Redmond WA, USA) SPOT, SPLASH, an MK10 (GPS) tags, Telonics, Inc. (Mesa, AZ, USA) ST‐6 and ST‐20 tags, and Sirtrack (Havelock North, New Zealand) KS‐101 tags (see Supporting Information Table S1.1 for number and location of tag deployments; all tags less than 1% of turtle body mass).

**Table 1:** Tagging locations and years for Kemp's ridley (*Lepidochelys kempii*) and loggerhead (*Caretta caretta*) sea turtles in the Gulf of Mexico

Tagging location	Kemp's ridley				Loggerhead				Total
	PAIS	RNMX	VCMX	GS	DRTO	GS	SJP	EAFB	
1998	1	.	.	.	.	.	.	.	1
2000	1	.	.	.	.	.	.	.	1
2004	2	.	.	.	.	.	.	.	2
2005	1	.	.	.	.	.	.	.	1
2006	3	.	.	.	.	.	.	.	3
2007	1	.	.	.	.	.	.	.	1
2008	1	.	.	.	2	.	.	.	3
2009	.	.	.	.	2	.	.	.	2
2010	4	1	.	.	1	.	4	.	10
2011	9	9	.	.	3	10	.	.	31
2012	8	.	2	1	3	7	6	2	29
2013	10	.	10	.	4	13	6	.	43
Total	41	10	12	1	15	30	16	2	127

Note

“.”: no turtles tagged for that location/year; DRTO: Dry Tortugas National Park, Florida; EAFB: Eglin Air Force Base, Florida; GS: Gulf Shores, Alabama; PAIS: Padre Island National Seashore, TX; RNMX: Rancho Nuevo, Tamalpais, Mexico; SJP: St. Joseph Peninsula, Florida; VCMX: Veracruz, Mexico.

Location data were retrieved using Satellite Tracking and Analysis Tool (STAT; Coyne & Godley, 2005) available on www.seaturtle.org (see Supporting Information Appendix S1 for general details on Location Class [LC] accuracy and Argos location processing). We retained LCs 3, 2, 1, 0, A, and B and filtered out LC Z for all analyses.

### 2.2 Determining foraging behavior with state‐space modeling

Switching state‐space modeling estimates location and behavioral mode at regular time intervals, accounting for satellite positional errors and dynamics of animal movement patterns (Jonsen, Flemming, & Myers, 2005). We conducted SSM following our previous studies (see Hart et al., 2014; Shaver et al., 2016 and Supporting Information Appendix S1 for additional information on this technique) to determine dates of each turtle at its foraging ground(s). The behavioral mode was binary, defined as “migration” or “foraging” in earlier applications (Breed, Jonsen, Myers, Bowen, & Leonard, 2009; Jonsen et al., 2005; Jonsen, Myers, & James, 2007); here, we deemed the modes “transiting” or “area‐restricted search” (ARS; Kareiva & Odell, 1987). Since we tagged animals during nesting seasons, we split ARS into “foraging” or “internesting” and considered “transiting” to be movements between these ARS modes. We considered foraging to occur after a migration, unless high‐quality locations on land indicated the turtle was still in the internesting period.

With the beginning and end dates for foraging periods determined by SSM, we used original, filtered satellite locations from within those time periods for further analysis. We filtered out locations on land, as well as those that were extremely distant or required movement speeds >5 kph. Kemp's ridleys primarily stay in waters −100 m deep or less (Fritts et al., 1983; Seney & Landry, 2011; Shaver & Rubio, 2008) and loggerheads generally stay within waters of the continental shelf (−200 m, Hawkes et al., 2011) so we also filtered out locations in waters deeper than −200 m (neritic zone cutoff, but see Supporting Information Appendix S1 for exceptions). Some turtles were recaptured and tagged twice. When two tracking periods were available, we used only the longest tracking period.

All Kemp's ridleys entered a migration mode, but two loggerheads did not, despite being tracked for more than 150 days (i.e., their foraging area was near their internesting area). We averaged the first month and day of the first foraging period for the other loggerhead turtles in order to calculate a mean foraging start date, 30 July, which we used as the beginning of the foraging period for these two turtles.

### 2.3 Home range comparisons and overlaps

We used both previously published (from Hart, Lamont, Fujisaki, Tucker, & Carthy, 2012; Hart et al., 2014; Shaver et al., 2013) and unpublished kernel density estimates (KDEs) and calculated new ones (KDE techniques follow our previous studies Hart et al., 2014; Shaver et al., 2016; Supporting Information Table S1.2 and Appendix S1 provide further details on analyses). In general, we used mean daily locations (when *N* ≥ 20) generated from filtered locations within the foraging area for KDE analysis. We used the home‐range tools for arcgis extension (Rodgers, Carr, Smith, & Kie, 2005) and fixed‐kernel least‐squares cross‐validation smoothing factor (*h*_cv_) for each KDE (Seaman & Powell, 1996; Worton, 1995). We used arcgis 9.3 (ESRI, 2007) to calculate the in‐water area (km^2^) within each kernel density contour (50% for core‐use and 95% for home range; Hooge, Eichenlaub, & Hooge, 2001) and to plot the data. Similar to KDEs in our previous studies, we also tested for site fidelity within foraging areas using the animal movement analysis extension for arcview 3.3 (ESRI, 2002; see Supporting Information Appendix S1). We bounded the range for random walks from −200 m to 0 m bathymetry to include only the realistic extent of the in‐water habitat for our animals during the study period; however, we smoothed out the shoreline with a 5 km buffer to account for many small bays and points close to land. We used a 10 km grid to show the number of individual KDEs per cell and where Kemp's ridleys and loggerhead KDEs co‐occurred. We also separated the loggerhead KDEs into their two nesting subpopulations to show any spatial overlap with where these turtles forage.

Further, to summarize where and when the two species co‐occurred, we determined how many 95% KDEs and individual turtles overlapped in both space and time. Overlaps in time were calculated using the entire date ranges of spatially co‐occurring 95% KDEs and counting any days of overlap, regardless of the percent of spatial overlap of the individual KDEs.

To characterize at‐sea foraging areas selected by individual turtles, we calculated the centroid of each turtle's 50% KDE as in our previous studies (see Supporting Information Appendix S1). We calculated the depth at each centroid and the distance from each centroid to the nearest land. For bathymetry, we used the NOAA National Geophysical Data Center (GEODAS) ETOPO1, 1 arc‐minute global relief model of Earth's surface (https://www.ngdc.noaa.gov/mgg/geodas/geodas.html; accessed 26 January 2012). Benthic habitat data are coarse for this region. We used a Dominant Sediments layer from the Gulf of Mexico Data Atlas (https://www.ncddc.noaa.gov/; accessed 20 February 2018) which provided dominant (>66% coverage) or subdominant (>33%) sediment types (sand, mud, gravel, or rock) for 0.02° (~4.5 km^2^) grid cells within the GoM. We summed the number of cells for each 95% KDE to determine the sediment type with the highest frequency for each KDE; we also assessed the species intersection in this way.

To compare core‐use areas and home ranges between Kemp's ridleys and loggerheads, we ran three Mann–Whitney rank sum tests on (a) 50% KDE areas; (b) 95% KDE areas; and (c) centroid distance to shore. For all of these tests, we included only one KDE per turtle; if a turtle had more than one KDE, only the last KDE/centroid was used.

### 2.4 Abiotic factors at home ranges

Environmental conditions are highly variable throughout the GoM. Although our previous studies highlighted foraging hot spots of Kemp's ridley (Shaver et al., 2013) and loggerheads in the GoM (Hart et al., 2014), it has been unclear whether the two species occupy similar habitat in a multidimensional space. To answer this question, we characterized the foraging location (50% KDE centroid) of each turtle using spatially explicit habitat data and we tested habitat similarity between the two species using permutational multivariate analysis of variance (MANOVA).

We derived mean, minimum, and maximum sea surface temperature (SST) for the period outside the nesting season for both species (September–March); raster data were derived from daily night SST, which were summarized by month of the year over multiple years and obtained from the Physical Oceanography Distributed Active Archive Center (PO.DAAC) at the NASA Jet Propulsion Laboratory, Pasadena, California (PO.DAAC, 2015; accessed 30 April 2014). We also derived mean net primary productivity (NPP) using Oregon State University ocean productivity data (https://www.science.oregonstate.edu/ocean.productivity; accessed 18 July 2014) during the same period. For SST and NPP, we used data between September 2011 and March 2014 during which most of the turtle‐tracking occurred.

As a preliminary analysis, we first conducted a Wilcoxon rank sum test to compare the two species with respect to six habitat variables including both spatial and environmental factors (latitude, longitude, distance to shoreline, SST, NPP, and bathymetry). This analysis was conducted to eliminate nonsignificant variables if there were any. We then visually examined a plot of the first two principal component scores using a set of selected variables based on the preliminary analysis. We used the “vegan” package of r (Oksanen et al., 2013) to conduct the permutational MANOVA.

We also conducted a Kruskal–Wallis test to compare foraging centroid bathymetry by tagging locations for each species. For each turtle, we used the first satellite location from the SSM input file as a proxy for the tagging locations. Exceptions were made if the first point was not near the known tagging location. If this was the case, the next point that was near the tagging location was used. For turtles with multiple KDEs, we used only the most recent KDE centroid, so that each turtle was represented by one centroid.

### 2.5 Ecoregion selection

The GoM is comprised of diverse marine ecoregions, each with unique ecological properties. We used foraging centroids for each turtle to determine their foraging ecoregion, based on the marine ecoregions delineated by Wilkinson et al. (2009). To understand the factors that dictated the selection of foraging regions of individuals, we used a categorical regression tree (CART) analysis to predict the foraging ecoregion of individual turtles from biotic (species and SCL) and spatial factors (tagging site).

## 3 RESULTS

We delineated foraging areas for 64 Kemp's ridleys and 63 loggerheads, totaling 127 turtles across 16 years (1998–2013). Kemp's ridleys ranged in size from 59.2 to 75.2 cm straight carapace length (SCL‐tip; mean ± *SD* = 63.5 ± 2.6 cm). Loggerheads ranged in size from 76.0 to 106.5 cm SCL‐tip (mean ± *SD* = 88.3 ± 5.4 cm; *N* = 13 loggerhead sizes were converted from CCLnt to SCLnt using the equation from Bjorndal et al., 2013). These turtles were tagged at various locations around the GoM, including in Mexico at Rancho Nuevo, Tamaulipas (RNMX, *N* = 10) and Veracruz (VCMX, *N* = 12), and in the United States at Padre Island National Seashore, Texas (PAIS, *N* = 41), Gulf Shores, Alabama (GS, *N* = 31), Eglin Air Force Base, Florida (EAFB, *N* = 2), St. Joseph Peninsula, Florida (SJP, *N* = 16), and Dry Tortugas National Park, Florida (DRTO, *N* = 15) (Table 1, Figure 2).

### 3.1 Home ranges

In total, we calculated 109 KDEs (58 for Kemp's ridleys and 51 for loggerheads) for 88 turtles (39 Kemp's ridleys and 49 loggerheads) (Table 2). We observed site fidelity for all foraging KDEs (proportions of random movement paths that were more randomly dispersed than constrained compared with turtle tracks ranged from >95.0495 to 100.0000 for each track). The KDEs included 5,460 foraging days with 3,939 mean daily locations for Kemp's ridleys and 7,580 foraging days with 4,714 mean daily locations for loggerheads (we did not always receive points every day that the turtles were in foraging mode; Supporting Information Table S1.3).

**Table 2:** Kernel density estimates (KDEs) for all foraging periods for Kemp's ridley (*Lepidochelys kempii*) and loggerhead (*Caretta caretta*) sea turtles in the Gulf of Mexico

		50% KDE (km^2^)	95% KDE (km^2^)	Centroid depth (m)	Centroid distance to shore (km)
Kemp's ridley *n* = 58 KDEs (39 turtles)	Min	10.6	51.8	−68.0	0.3
	Max	5,172.5	20,299.5	−1.0	93.9
	Mean	957.2	4,169.1	−16.2	22.9
	*SD*	1,154.8	4,876.5	13.5	19.0
Loggerhead *n* = 51 KDEs (49 turtles)	Min	4.5	22.0	−72.0	0.6
	Max	851.8	3,628.5	0.0	246.0
	Mean	83.6	430.2	−28.3	55.0
	*SD*	129.7	571.5	20.7	56.2

Note

Centroid depth: the bathymetry measurement at the site of the centroid.

50% KDEs represent core‐use areas and 95% KDEs represent the home range. Foraging periods were not combined and counted separately for turtles with more than one. Centroids were created from 50% KDEs.

**Figure 3:**
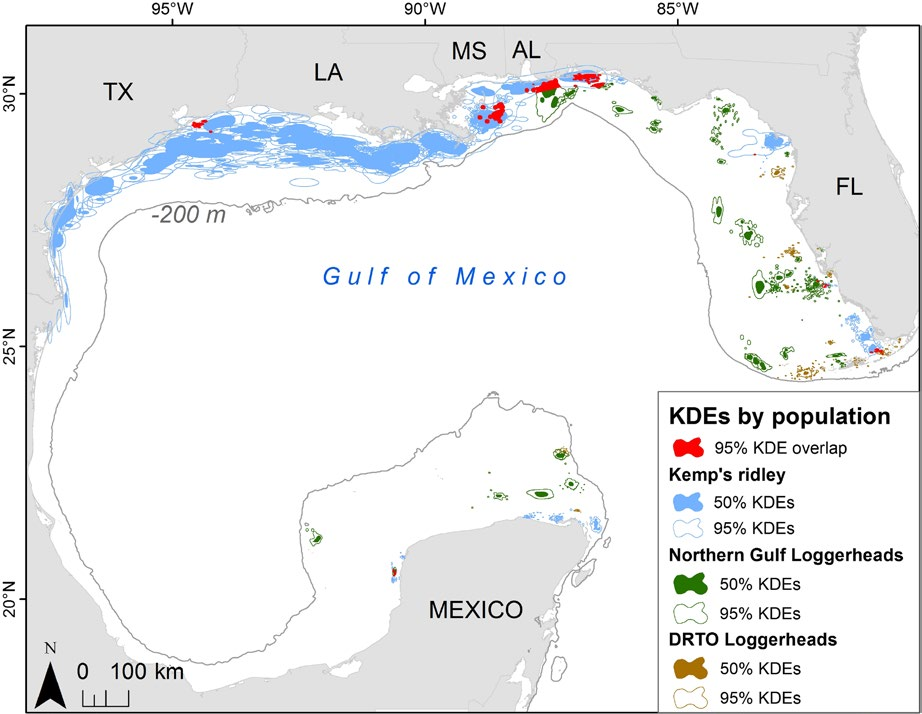
Kernel density estimates (KDEs) for 39 Kemp's ridley (*Lepidochelys kempii*; 58 KDEs) and 49 loggerheads (*Caretta caretta; *51 KDEs color‐coded based on subpopulation/tagging location) sea turtles tagged in the Gulf of Mexico. 50% KDEs represent core‐use areas and 95% KDEs represent home ranges. Red areas highlight where home ranges co‐occur for the two species. DRTO: Dry Tortugas National Park

Mean values for core‐use areas (50% KDEs) were about eleven times larger for Kemp's ridleys (mean 957.2 km^2^) than for loggerheads (mean 83.6 km^2^; Table 2 and Figure 3). The home ranges (95% KDEs) followed a similar pattern, with Kemp's ridley home ranges (95% KDE mean 4,169.1 km^2^) almost ten times larger than loggerhead home ranges (95% KDE mean 430.2 km^2^; Table 2 and Figure 3). Core‐use areas (50% KDEs) were significantly larger for Kemp's ridleys than loggerheads (Mann–Whitney *U* = 368.0, *p* < 0.001), and home ranges (95% KDEs) followed the same pattern (Mann–Whitney *U* = 341.0, *p* < 0.001).

### 3.2 Co‐occurrence of foraging home ranges

The 95% KDEs of both species showed spatial overlap throughout the GoM, including off the coasts of all U.S. Gulf states, as well as on the western shore of the Yucatán Peninsula (Figure 3). The co‐occurrence areas included 11 individual loggerheads (12 KDEs) and 16 Kemp's ridleys (20 KDEs) for a total of 28 unique Kemp's/loggerhead co‐occurrence combinations. However, these areas were small in comparison with the widespread species' distributions. Combined, the co‐occurrence areas totaled 2,850.0 km^2^, only 1.1% of the sum total 95% KDE area for all turtles (21,942 km^2^ for loggerheads, 241,806 km^2^ for Kemp's).

**Figure 4:**
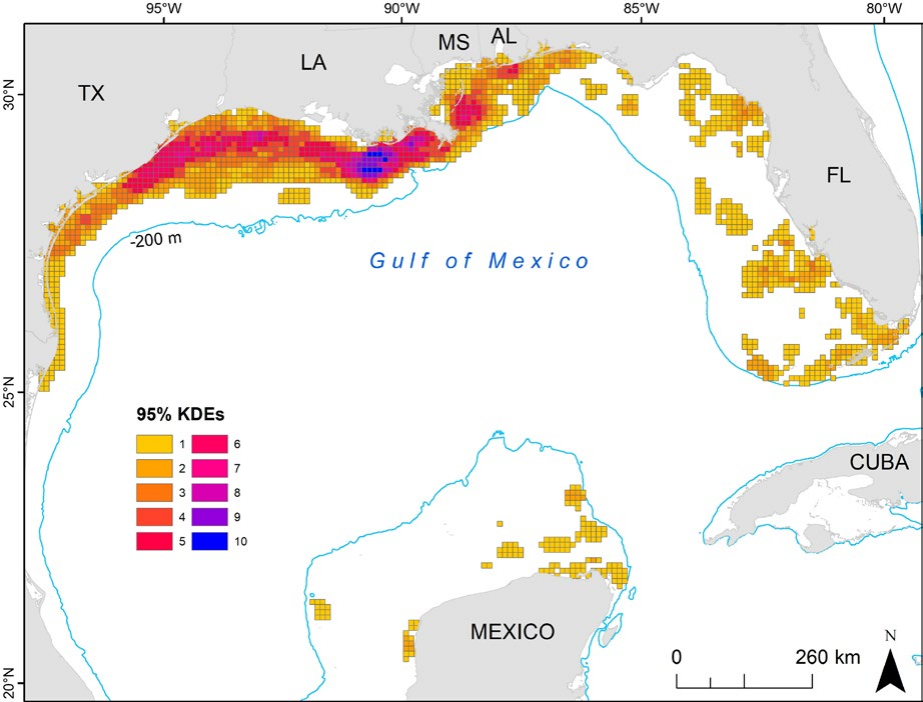
The number of individual turtles with home ranges (95% Kernel Density Estimates [KDEs]) in 10 km grid cells throughout the Gulf of Mexico. Both Kemp's ridley (*Lepidochelys kempii*) and loggerhead (*Caretta caretta*) KDEs are included: If a turtle had more than one KDE, these were merged so that every turtle was only counted once throughout all grid cells

**Figure 5:**
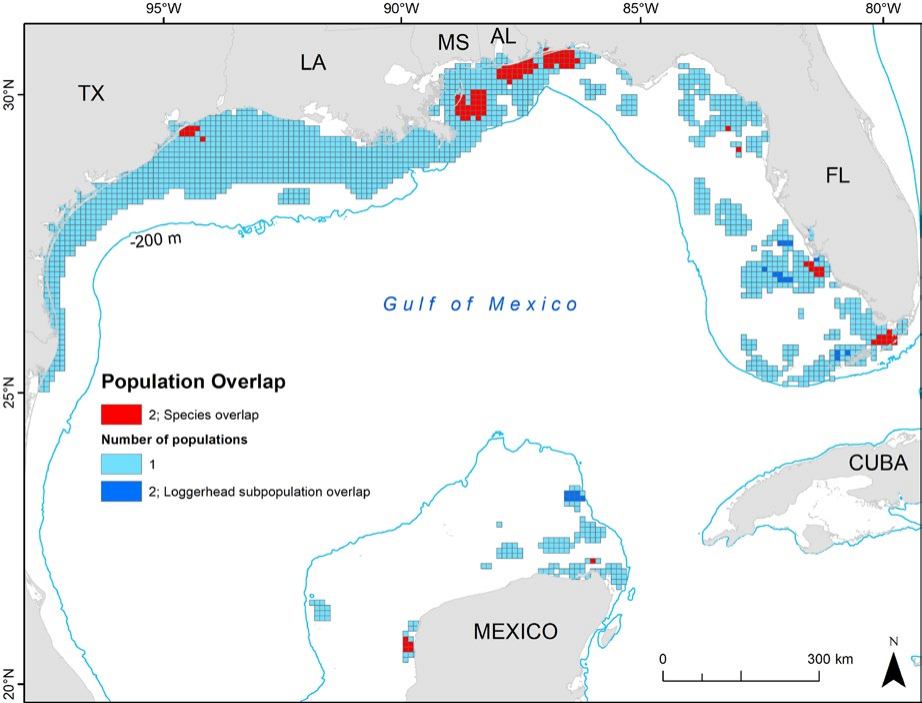
The number of subpopulations with intersecting home ranges (95% Kernel Density Estimates) in each 10 km grid cell throughout the Gulf of Mexico: Turtles from the same subpopulation were combined and could include loggerheads (*Caretta caretta*) tagged in Dry Tortugas National Park, loggerheads tagged in the Northern Gulf, or Kemp's ridleys (*Lepidochelys kempii*), for a possible maximum of three subpopulations represented per grid cell. However, we did not find all three in any grid cell. Therefore, dark blue cells indicate where the two loggerhead subpopulations co‐occur, and red indicates where Kemp's ridleys co‐occur with a single loggerhead subpopulation

The 10‐km grid cells highlight neritic waters off the Louisiana coast as having a higher concentration of individual 95% KDEs co‐occurring (primarily for Kemp's ridleys, Figure 4) with multiple locations throughout the GoM serving as co‐occurring foraging areas for two loggerhead subpopulations and/or the two species (Figure 5). The two species overlap occurred in 10,200 km^2^ of gridded area, about 6% of the entire grid area where at least one turtle's 95% KDE occurred.

Of the 28 KDEs that overlapped in space, there were six unique Kemp's ridley/loggerhead co‐occurrence combinations that also overlapped in time. These occurred in years 2010–2013 and months July‐November (Table 3). For these combinations, the time each KDE overlapped ranged from 2 to 94 days (mean ± *SD* = 34.3 ± 32.5 days) for a total of 206 days across all combinations. These six unique space and time co‐occurrence areas ranged in size from 18 to 191 km^2^ (mean ± *SD* = 78.6 ± 73.0 km^2^) for a total of 471.7 km^2^. Looking at each turtle pair that showed some overlap, these space and time co‐occurrences represented only a portion of the two home ranges involved: 0.1%–12.8% (mean ± *SD* = 3.9% ± 4.6%; Table 3). The combination with the largest percent overlap in area had the second smallest overlap in time (11 days).

**Table 3:** Home range (95% Kernel Density Estimate [KDE]) spatial and temporal overlap for Kemp's ridley (*Lepidochelys kempii*) and loggerhead (*Caretta caretta*) sea turtles tagged in the Gulf of Mexico

Loggerhead			Kemp's ridley			Overlap		
95% KDE	Dates	Area (km^2^)	95% KDE	Dates	Area (km^2^)	Dates (days)	Area (km^2^)	Percent area (km^2^) overlap
106,361	7/1/11–6/18/12	3,628.5	100,394	8/8/11–11/9/11	725.2	8/8/11–11/9/11(94)	150.9	3.5
119,942	9/4/12–9/29/12	893.5	101,138	9/3/12–10/30/12	475.6	9/4/12–9/29/12(26)	45.7	3.3
129,513	7/22/13–8/12/13	238.1	112,766	6/13/13–7/23/13	20,299.5	7/22/13–7/23/13(2)	17.6	0.1
129,512	7/26/13–10/15/13	194.8	112,766	9/6/13–10/7/13	17,372.1	9/6/13–10/7/13(32)	190.6	1.1
52,958	8/21/10–9/29/11	1,037.6	47,524	9/9/10–10/19/10	117.4	9/9/10–10/19/10	31.7	2.7
129,507	9/6/13–10/1513	106.3	126,234	6/25/13–9/16/13	168.5	9/6/13–9/16/13(11)	35.2	12.8
					Mean	34.3	78.6	3.9
					*SD*	32.5206	73	4.6
					Sum		471.7	

Note

Each line represents KDEs that overlapped in both space and time; turtle ID (95% KDE column), dates and area of the KDE are provided for the loggerhead, Kemp's ridley separately, and then the area of overlap.

Sediment‐type grid cells were available for 42/51 loggerhead KDEs (overlapping coverage of sediment and KDE layers at 63%–100% for KDEs; mean 97%) and 52/58 Kemp's ridley KDEs (coverage of 69%–100% at KDEs; mean 97%; Supporting Information Tables S1.4 and S1.5). For most loggerhead KDEs (33/42; 79%), the highest number of grid cells was defined as being dominant or subdominant for sand. For most Kemp's ridley KDEs (35/52; 67%), the highest number of grid cells was defined as dominant for mud. The majority of grid cells (566/946; 60%) for the area of intersection (96% coverage) was classified as dominant or subdominant for sand. The next highest count (308/946; 33%) of cells was dominant or subdominant for mud.

### 3.3 Habitat similarity and ecoregion selection

Loggerhead foraging centroids were in deeper water on average (−28.3 m; *SD*: 20.7 m) than Kemp's ridley centroids (−16.2 m; *SD*: 13.5 m) (Table 2). Mean distance from shore for loggerhead centroids was 55.0 km, which was twice as far as Kemp's ridley foraging centroids (22.9 km; Table 2). When we compared bathymetry at foraging centroids of Kemp's ridleys that nested in Texas (PAIS, *N* = 29) and Mexico beaches (Rancho Nuevo and Veracruz, *N* = 10), the difference was not significant (Kruskal–Wallis *χ* ^2^ = 0.150, *p* = 0.699). However, bathymetry at foraging sites for loggerheads tagged in three areas (GSAL and EAFB, PSJ, and DRTO) was significantly different (Kruskal–Wallis *χ* ^2^ = 7.03, *p* = 0.030); the mean foraging centroid bathymetry for these three tagging locations is −33.96 m, −27.8 m, and −16.67 m, respectively.

**Figure 6:**
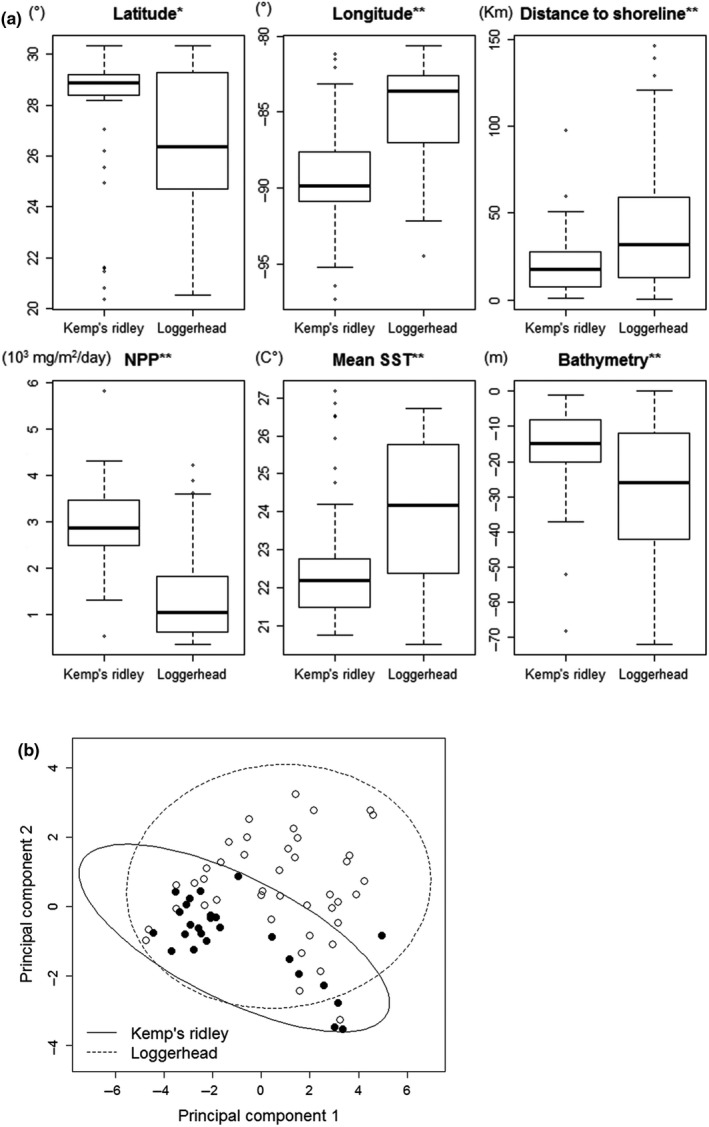
(a) Box plots of six habitat variables (latitude, longitude, distance to shoreline, daytime mean sea surface temperature, and net primary production) at an identified foraging location (centroid of 50% Kernel Density Estimate [KDE]) of 88 nesting turtles (39 Kemp's ridleys [*Lepidochelys kempii*] and 49 loggerheads [*Caretta caretta*]) satellite‐tracked in the Gulf of Mexico. Note other sea surface temperature variables used for comparing the habitat similarity are not shown because they are correlated with the mean SST. Star marks indicate that species difference was significant (* <0.05, ** <0.01). All variables that were significant at an *α*‐level of 0.01 were also significant after the Bonferroni correction. (b) The scatter plot of first two principal component scores calculated from 11 habitat variables (latitude, longitude, daytime and nighttime mean, minimum, and maximum sea surface temperatures, net primary production, bathymetry, and distance to the shoreline) at an identified foraging location (centroid of 50% KDE) of 88 nesting turtles (39 Kemp's ridleys [black dots] and 49 loggerheads [open circles]) satellite‐tracked in Gulf of Mexico. The polygons indicate 95% confidence ellipses.

Final foraging sites for Kemps' ridley and loggerheads differed significantly in all spatial and environmental variables according to the Wilcoxon rank sum tests (*p* < 0.05 for all; Figure 6a); therefore, we included all variables in the analysis. There were some moderately high to high bivariate correlations between habitat variables, including correlations between latitude and SST variables (|*r*| > 0.9) and between NPP, bathymetry, and distance to shoreline (|*r*| > 0.6). The first two principal component (PC) scores from all habitat variables explained almost 100% of variability contained in the data. The scatter plots of the two PC scores indicated a considerable overlap in ecological niche space between the two species, yet overall loggerheads occupied a larger niche space that also contained the niche space occupied by Kemp's ridleys (Figure 6b). The results of the permutational MANOVA (*F* = 12.951, *p* = 0.0001) indicated habitat variables at the final foraging sites of the two species are significantly different.

**Figure 7:**
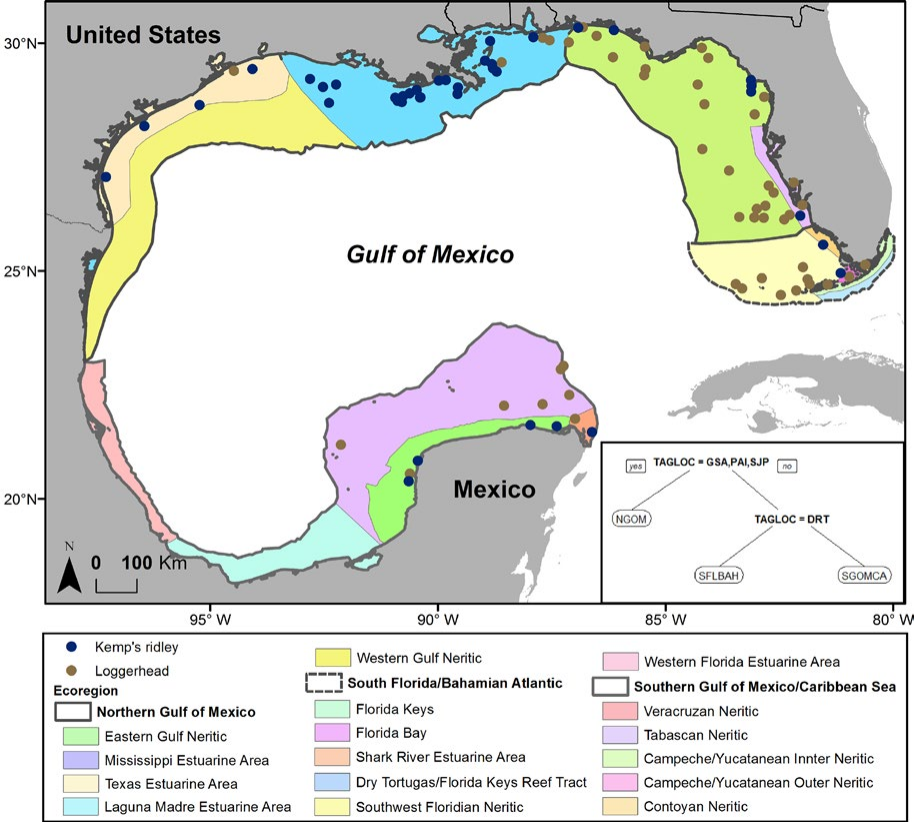
Identified final foraging locations (centroid of 50% Kernel Density Estimate [KDE]) of 88 nesting turtles (39 Kemp's ridleys [*Lepidochelys kempii*] and 49 loggerheads [*Caretta caretta*]) satellite‐tracked in the Gulf of Mexico. The ecoregions were hand‐digitized based on coastal regions in Wilkinson et al. (2009). Inset: Final model of categorical regression tree to predict foraging ecoregions (NGOM: Northern Gulf of Mexico; SFLBAH: South Florida/Bahamian Atlantic; SGOMCA: Southern Gulf of Mexico/Caribbean Sea) from nesting area (DRTO: Dry Tortugas National Park; GS: Gulf Shores; PAI: Padre Island National Seashore; SJP: St. Joseph Peninsula)

Final foraging sites of the 88 turtles were distributed in 12 level III (finer scale) ecoregions in the GoM (Figure 7). On a coarser level (level II), 13 turtles went to South Florida/Bahamian Atlantic (two Kemp's ridleys and 11 loggerheads), 64 turtles went to Northern Gulf of Mexico (32 Kemp's ridleys and 30 loggerheads), and 13 turtles went to southern Gulf of Mexico/Caribbean Sea (five Kemp's ridleys and eight loggerheads). The CART analysis showed that capture site is the single dominant variable that dictates final foraging ecoregion of turtles (Figure 7), accounting for 66% of variable importance followed by SCL (19%) and species (17%).

## 4 DISCUSSION

Recent meta‐analyses have found that species co‐occurrence is generally less than expected by chance, although presence–absence matrices specifically for herpetofauna found less structure than for homeotherms (Gotelli & McCabe, 2002; Ulrich & Gotelli, 2010). For decades, it has been known that Kemp's ridley and loggerhead turtles co‐occur throughout their range in the Gulf of Mexico (Fritts et al., 1983; Hildebrand, 1982; Márquez, 1990; Rabalais & Rabalais, 1980) and that both species inhabit and forage in nearshore waters (Lewison, Crowder, & Shaver, 2003; Márquez, 1994; Plotkin, Wicksten, & Amos, 1993; Shaver, 1991). We found areas of co‐occurrence existed for two sea turtle species in GoM neritic waters, but the majority of foraging KDEs were located in separate locations. Kemp's ridley foraging home ranges were concentrated in the northern GoM, whereas loggerhead foraging home ranges were concentrated in the eastern GoM. In addition, areas where the two turtle species co‐occurred made up a small proportion of the total home range area (1.1%) and for the six unique KDE combinations that co‐occurred in both space and time, co‐occurrence was minimal (mean 3.9% area overlap and mean 34.3 days). Our results fall in line with the meta‐analyses, as we did not find a high degree of species overlap.

Factors determining the co‐occurrence (or lack thereof) of species in natural communities have long been a subject of debate. Diamond (1975) introduced “assembly rules” that considered competition to be a major driving force of species distributions. However, these rules have been challenged as null models were able to predict similar habitat distributions in the absence of interspecific competition (Connor & Simberloff, 1979; Gotelli, 1999), raising more questions on what drives the co‐occurrence of species. Further complicating this, competition may be important at some spatial scales and not others, with the importance potentially waning at scales of about 1,000 km by 500 km (McGill, 2010)—similar to the scale of the GoM. Little is known about interspecific sea turtle interactions and how they may influence distributions. Whether competition plays a large role in determining sea turtle distribution at any scale is not well known. To understand the scale at which competition may be important, it is necessary to have data on other factors that may influence species distributions (McGill, 2010), such as prey resources, dispersal and environmental conditions.

### 4.1 Prey resources

Although studies have shown that Kemp's may forage opportunistically (Witzell & Schmid, 2005), those studies focused almost exclusively on immature Kemp's ridleys and differences may occur among life‐stages with adults becoming more specialized on crab consumption (Shaver, 1991). Foraging adult and subadult Kemp's ridleys in south Texas waters were found to eat primarily bottom‐dwelling or swimming crabs (*Callinectes sapidus* and *Areaneus cribrarius*; Shaver, 1991). Shaver (1991) suggested that Kemp's ridleys forage within −50 m water depth based on gut contents and the distribution of crabs in the GoM: Both of these species are common in the northern GoM and found almost exclusively in shallow waters less than 70 m deep (Powers, 1977). Our foraging centroid locations support this assertion, as only 2 out of the 39 Kemp's ridley centroids occurred in water deeper than −50 m (Turtle 100,403: −52 m, Turtle 104,404: −68 m) and the average water depth for centroids was shallower than −20 m. Loggerhead centroids were on average in water deeper than for Kemp's ridleys (mean −28.3 m). Loggerheads also forage on crabs, but they are considered generalists (Vander Zanden, Bjorndal, Reich, & Bolten, 2010) therefore they may utilize deeper water to forage on species such as whelks or fish. Additionally, Kemp's ridley home ranges were significantly and substantially (~10×) larger than loggerhead home ranges. The wider breadth of prey on which loggerheads forage may allow them to remain in a narrower area, whereas Kemp's ridleys may search for their specific prey item resulting in a larger home range than loggerheads.

Another consideration in reviewing the prey resources of these species is that the northeastern GoM represents a dividing line for population genetics of various taxa including blue crabs (Apalachicola River; Darden, 2004) and other fish and invertebrates (Mobile Bay, Alabama; Portnoy & Gold, 2012). The area from Mobile Bay to the Apalachicola River is also where the majority of KDEs for Kemp's ridleys and loggerheads showed a geographic split. Immature Kemp's ridleys are found throughout the GoM (Eaton et al., 2008), indicating that crabs may play an important role in regulating adult distribution (Shaver, 1991).

### 4.2 Dispersal effects

We found that the nesting beach was the most important variable we tested in predicting foraging ecoregion for turtles in the GoM, suggesting that dispersal plays an important role in turtle distribution. Loggerheads have been documented to travel hundreds or thousands of kilometers from nesting beaches to foraging grounds; a summary of loggerhead migration distances in the Atlantic and Mediterranean showed a mean distance of 618 km, with a maximum of 2,150 km (Hays & Scott, 2013), which exceeds the lengths of the U.S. Gulf of Mexico axes (~1,000 km by ~1,500 km). The densest loggerhead nesting beaches in the GoM are found on beaches along Florida's west coast in the eastern GoM; west of Florida only sporadic loggerhead nesting occurs in Louisiana and Texas (NMFS & U.S. Fish & Wildlife Service [USFWS], 2008). Additionally, few tracked loggerheads have moved into the western GoM postnesting (Foley, Schroeder, Hardy, MacPherson, & Nicholas, 2014; Girard, Tucker, & Calmettes, 2009; Hart et al., 2014). This may be due to plentiful resources for loggerheads in the eastern GoM or perhaps this represents the dispersal limit for these subpopulations in the GoM.

Kemp's ridleys only occur within GoM and Atlantic waters and individuals have been tracked migrating along the entire U.S. GoM shoreline (~1,600 km) and from Mexican tagging locations out to the Yucatán Peninsula (Shaver et al., 2016). Based on previous SSM work in the GoM, loggerheads generally have clearer distinctions between migration and foraging modes as compared to Kemp's ridleys that show continual switching between migration and foraging modes as they move along the GoM coast (Hart, Lamont, et al., 2012; Shaver et al., 2013). Perhaps dispersal limitations are more important for loggerheads that do not repeatedly interrupt migration to replenish energy reserves. Additionally, dispersal (i.e., migration) to and from nesting grounds may impact temporal co‐occurrence on foraging grounds. Kemp's ridleys complete their annual nesting season earlier than loggerheads (July vs. August, respectively), so the timing of Kemp's arrival at the foraging grounds would be expected to occur earlier than loggerheads.

The distribution of sea turtle foraging areas in the Mediterranean is influenced strongly by the timing and distance of hatchling dispersal based on passive drift (Hays, Fossette, Katselidis, Mariani, & Schofield, 2010), and this type of dispersal may also play a role in the patterns we observed in the GoM. However, genetic evidence demonstrated that directed swimming by surface‐pelagic juvenile green turtles in the GoM, and not just passive drift, contributed to their ultimate foraging destinations (Shamblin, Witherington, Hirama, Hardy, & Nairn, 2018). Additionally, experiments that deployed surface drifters alongside both green and Kemp's ridley juveniles showed that these turtles were not passively drifting but actively swimming (Putman & Mansfield, 2015). For loggerheads and Kemp's ridleys in the GoM, hatchling and small juvenile dispersal could influence foraging site selection and neritic recruitment, resulting in divergence in site selection between the species for reasons that have less to do with resource availability and use and more with ontogeny. Future comparisons of both loggerhead and Kemp's ridley postnesting females from Mexico could help determine to what degree ontogeny plays a role in the choice of foraging areas between the two species.

### 4.3 Environmental effects

Establishing important environmental factors for foraging habitat can help explain species distribution (the fundamental niche; Hutchinson, 1957; Soberón, 2007) as well as provide a baseline for detecting shifts in distribution based on changes in, for example, water temperature and depth that may be brought on due to climate change. We found that Kemp's ridley and loggerhead foraging sites showed differences in all six spatial and environmental variables we tested (latitude, longitude, distance to shoreline, SST, NPP, and bathymetry). Loggerheads, perhaps due to their generalist foraging behavior, showed a wider niche space than Kemp's ridleys and also overlapped the Kemp's ridley niche space. This suggests that Kemp's ridleys may be more vulnerable to environmental changes and that loggerheads could theoretically occupy Kemp's ridley foraging areas, even though empirically we saw a division of habitat‐use.

### 4.4 Spatial dynamics

A metacommunity can be defined as a group of local communities with interacting species linked by dispersal (Leibold, et al., 2004). While we primarily saw loggerheads and Kemp's ridleys on east and west sides of the GoM respectively, a few individuals did forage on “opposite” ends, indicating that they can be linked by dispersal and that appropriate “patches” occur across the GoM for both species. Interaction in the foraging areas is also plausible because of their similar dietary requirements and some empirical evidence of spatial overlap. The species sorting perspective of metacommunity theory states that environmental heterogeneity influences species responses and that habitat (i.e., patch) quality affects local composition in combination with dispersal (Leibold, et al., 2004). We are drawing a link between turtle presence and assumed community type based on general knowledge of their spatial locations and similar ecological roles in the GoM. That community types occupied by each species may be ecologically similar for turtles is also supported by their niche space overlaps. However, we did find that environmental variables were an important factor in determining occurrence across foraging sites, indicating patch quality may influence local composition. Only limited, coarse‐scale benthic habitat data are available for these foraging areas (i.e., “mud,” “sand,” “gravel,” or “rock”); however, we saw evidence that there may be differences in patch quality for the different species such that loggerheads may prefer sand while Kemp's ridleys may prefer mud. We feel that this example involving marine megafauna represents one of a few empirical tests of this organizing concept. Additionally, the few areas of overlap may provide sites with a heterogenous environment allowing for co‐occurrence and further habitat studies in these areas are warranted. We observed this at least for sediment types, with the intersect area having both sand and mud. Future habitat modeling with finer‐scale benthic resolution as well as prey abundance surveys would be valuable for understanding habitat differences for these species. Regardless, these areas represent clear long‐term and persistent locations of high‐use zones for key imperiled species.

## 5 CONCLUSION

We build on previous work delineating foraging areas for Kemp's ridleys and two subpopulations of loggerheads across seven nesting sites in the GoM and demonstrate locations where two imperiled sea turtle species co‐occur during their foraging periods. However, our analysis was restricted to adult females. Determining habitat‐use and whether similar drivers affect the distribution for males and juveniles will be important for the conservation of these species.

Unless habitats and dispersal for two species are similar, parsing out the importance of competition may not be possible, as distribution may be due more to changes in habitat or dispersal ability (Ulrich & Gotelli, 2013). It is possible that Kemp's ridleys and loggerheads may compete for resources; both species have been documented to forage extensively on crabs (Burke, Standora, & Morreale, 1993; Wallace, Avens, Braun‐McNeill, & McClellan, 2009). However, the separation in foraging areas (based on centroid locations and low level of direct overlap), the significant differences in environmental conditions at foraging grounds, and the importance of nesting location on ecoregion selection (i.e., dispersal ability) indicate that adult females of these species do not interact greatly during foraging, that they show little syntopy, and that dispersal and environmental factors more strongly determine their distributions within their shared GoM region.

Analyses on plant and mammal communities across geological time have shown that aggregated species were more common before the increase in human population during the Holocene, suggesting that current evidence for sympatry and syntopy may also be impacted by human activity (Lyons et al., 2016). As these two sea turtle species evolved about 3–6 million years before this Holocene shift (Bowen, Meylan, & Avise, 1991), it is possible that the distributions we see today might be more constricted than in the past. Anthropogenic impacts in the GoM are ongoing and across 19 modeled stressors, the highest were for ocean acidification, sea surface temperature, sea level rise, ultraviolet anomalies, pollution, and shipping (for 2013; Halpern et al., 2015). Therefore, continuing to investigate how anthropogenic pressures influence species' habitat selection will be a key addition to understanding drivers of distribution for these imperiled sea turtles.

## CONFLICT OF INTEREST

None declared.

## AUTHOR CONTRIBUTIONS

K. H., D. S., and A. I. conceived the ideas; K. H., M. L., and D. S. collected the data; A. I., I. F., and D. B. analyzed the data; K. H. and A. I. led the writing. All authors contributed critically to the drafts and gave final approval for publication.

## DATA ACCESSIBILITY

Raw data are exempt from publication due to sensitivity of endangered species location information. All other data used for analyses are presented in the paper or in the Supporting Information Appendix S1.
