# Supplementary material for: Sympatry or syntopy? Investigating drivers of distribution and co‐occurrence for two imperiled sea turtle species in Gulf of Mexico neritic waters
Source: Ecol Evol. 2018 Nov 26;8(24):12656–69. doi: 10.1002/ece3.4691 (PMC6308884; doi:10.1002/ece3.4691)
Supplement: Supplementary file 1 [file ECE3-8-12656-s001.docx]

*Ecology and Evolution*

**SUPPORTING INFORMATION**

**Sympatry or syntopy?: Investigating drivers of distribution and co-occurrence for two imperiled sea turtle species in Gulf of Mexico neritic waters**

Kristen M. Hart, Autumn R. Iverson, Ikuko Fujisaki, Margaret M. Lamont, David Bucklin, and Donna J. Shaver ^e^

**Appendix S1** Sea turtle tagging, tracking, and analysis details.

Tags were programmed variously for the turtles:

*Loggerheads* (*Caretta caretta*)

2008-2010: continuously

2011-2013: continuously then every 3^rd^ day November-April

*Kemp’s ridleys* (*Lepidochelys kempii*)

1998-2007: 6 h on/6 h off

2008: continuously for 106 days, then 6 h on/6 h off

2010-2013: either continuously or 6 h on/6 h off

Argos assigns these accuracy estimates for location classes (LCs; CLS, 2011):

LC 3: <250 m

LC 2: 250 to <500 m

LC 1: 500 to <1500 m

LC 0: >1500 m

LC A: unknown

LC B: unknown

LC Z: failed Argos plausibility tests

From 1998-2010, Argos performed traditional least-squares location processing. In 2011, Argos initiated Kalman-filtering (Kalman, 1960) on location data. The Kalman-filtering algorithm provides more estimated positions and significantly improves position accuracy, especially for LCs A and B (Lopez, Malarde, Royer, & Gaspar, 2013).

**Switching state space modeling (SSM)**

We used SSM (Jonsen, Myers, & Flemming, 2003; Patterson, Thomas, Wilcox, Ovaskainen, & Matthiopoulos, 2008) to characterize the movements of all turtles. Jonsen, Flemming, & Myers (2005) described the model and since then it has been applied to marine animals including turtles (Bailey et al., 2008; Hart, Lamont, Fujisaki, Tucker, & Carthy, 2012; Hart, Lamont, Sartain, Fujisaki, & Stephens, 2013; Hoenner, Whiting, Hindell, & McMahon, 2012; Jonsen, Myers, & James, 2006; Jonsen, Myers, & James, 2007; Maxwell et al., 2011; Shaver et al., 2013). Satellite-transmitted location data are often received at irregular time intervals, and can sometimes have large gaps and positional errors. Filtering by LC is a first step, but it does not remove all erroneous locations and can result in loss of information (Jonsen et al., 2006). We followed Breed, Jonsen, Myers, Bowen, & Leonard (2009) and applied a model that is a modified version of a model described by Jonsen et al. (2005). We estimated model parameters by Markov Chain Monte Carlo (MCMC) using WinBUGS via R with package R2WinBUGS (Sturtz, Ligges, & Gelman, 2005). We fit the model to tracks of each individual turtle and estimated the behavioral mode and location of the turtles every eight hours during the tracking period. We used two independent and parallel chains of MCMC. Our samples from the posterior distribution were based on 10,000 iterations after a burn-in of 7000 and thinned by five.

**Foraging Areas**

*KDEs*

Some kernel density estimates (KDEs) are from previously published papers or unpublished sources. See Table S1.2 for the full list. We used the filtered locations within the foraging area to generate mean daily locations. This minimized autocorrelation and the resulting coordinates provided raw data for KDE analysis. When we observed unequal variance of the x and y coordinates, we followed previous studies and rescaled the data to select the best bandwidth (Laver and Kelly, 2008; Seaman and Powell, 1996).

*Site Fidelity*

Using Monte Carlo Random Walk simulations (100 replicates), we tested tracks during a turtle’s time at the foraging ground for spatial randomness against randomly generated walks (Hooge, Eichenlaub, & Hooge, 2001). Tracks exhibiting site fidelity indicate movements that are more spatially constrained rather than randomly dispersed (Hooge et al. 2001).

*Centroids*

We generated centroid locations for each 50% KDE using ArcGIS 9.3 (ESRI, 2007). If a 50% KDE included multiple activity centers, we calculated the centroid of the largest activity center.

**Depth filtering and bounding polygon exceptions**

Unpublished summaries in 2013 and 2014 for kernel density estimates from switching state-space modeling (all Kemp’s ridley turtles) match the current methods except for these two small deviations:

1. For both the 2013 and 2014 summaries, we filtered points using a depth cut-off of -100 m, as is generally accepted for Kemp’s ridley turtles (see Methods).
2. Also, for the random walks to determine site fidelity, we bounded the points in these summaries from -100 m to 0 m bathymetry to include only the realistic extent of the in-water habitat for our animals during the study period. Similarly to the current methods, we smoothed out the North Gulf shoreline with a buffer to account for many small bays and points close to land, however this buffer was created at 2 km instead of 5 km.

**REFERENCES**

Bailey, H., Shillinger, G., Palacios, D., Bograd, S., Spotila, J., Paladino, F., & Block, B. (2008). Identifying and comparing phases of movement by leatherback turtles using state-space models. Journal of Experimental Marine Biology and Ecology, 356, 128–135.

Breed, G.A., Jonsen, I.D., Myers, R.A., Bowen, W.D., & Leonard, M.L. (2009). Sex-specific, seasonal foraging tactics of adult grey seals (*Halichoerus grypus*) revealed by state-space analysis. *Ecology,* 90, 3209–3221.

CLS. (2011). Argos user’s manual: worldwide tracking and environmental monitoring by satellite. 19 August 2011 update. Retrieved from: http://www.argossystem.org/web/en/76-user-s-manual.php.

Environmental Systems Research Institute (ESRI). (2007). ArcGIS 9.3 GIS. Redlands, CA.

Hart, K.M., Lamont, M.M., Fujisaki, I., Tucker, A.D., & Carthy, R.R. (2012). Common coastal foraging areas for loggerheads in the Gulf of Mexico: Opportunities for marine conservation. *Biological Conservation*, 145, 185–194.

Hart, K.M., Lamont, M.M., Sartain, A.R., Fujisaki, I., & Stephens, B.S. (2013). Movements and habitat-use of loggerhead sea turtles in the Northern Gulf of Mexico during the reproductive period. *PLoS One*, 8, e66921.

Hoenner, X., Whiting, S.D., Hindell, M.A., & McMahon, C.R. (2012). Enhancing the use of Argos satellite data for home range and long distance migration studies of marine animals. *PLoS One*, 7, e40713.

Hooge, P.N., Eichenlaub, W., & Hooge, E.R. (2001). Animal movement 2.5. US Geological Survey, Anchorage, AK.

Jonsen, I.D., Myers, R.A., & Flemming, J.M. (2003). Meta-analysis of animal movement using state-space models. *Ecology*, 84, 3055–3063.

Jonsen, I.D., Flemming, J.M., & Myers, R.A. (2005). Robust state-space modeling of animal movement data. *Ecology*, 86, 2874–2880.

Jonsen, I.D., Myers, R.A., & James, M.C. (2006). Robust hierarchical state-space models reveal diel variation in travel rates of migrating leatherback turtles. *Journal of Animal Ecology*, 75, 1046–1057.

Jonsen, I.D., Myers, R.A., & James, M.C. (2007). Identifying leatherback turtle foraging behaviour from satellite telemetry using a switching state-space model. *Marine Ecology Progress Series*, 337, 255–264.

Kalman, R.E. (1960). A new approach to linear filtering and prediction problems. *Journal of Basic Engineering*, 82, 35–45.

Laver, P.N., & Kelly, M.J. (2008). A critical review of home range studies. *The Journal of Wildlife Management*, 72, 290–298.

Lopez, R., Malarde, J., Royer, F. & Gaspar, P. (2013) Improving Argos doppler location using multiple-model kalman filtering. *Geoscience and Remote Sensing, IEEE Transactions on*, 52, 4744–4755.

Maxwell, S.M., Breed, G.A., Nickel, B.A., Makanga-Bahouna, J., Pemo-Makaya, E., Parnell, R.J., … & Coyne, M.S. (2011). Using satellite tracking to optimize protection of long-lived marine species: olive ridley sea turtle conservation in Central Africa. *PLoS One*, 6, e19905.

Patterson, T.A., Thomas, L., Wilcox, C., Ovaskainen, O., & Matthiopoulos, J. (2008) State-space models of individual animal movement. Trends in Ecology & Evolution, 23, 87–94.

Seaman, D.E., & Powell, R.A. (1996). An evaluation of the accuracy of kernel density estimators for home range analysis. *Ecology*, 77, 2075–2085.

Shaver, D.J., Hart, K.M., Fujisaki, I., Rubio, C., Sartain, A.R., Peña, J., … & Ortiz, J. (2013) Foraging area fidelity for Kemp’s ridleys in the Gulf of Mexico. *Ecology and Evolution*, 3, 2002–2012.

Sturtz, S., Ligges, U., & Gelman, A. (2005) R2WinBUGS: A package for running WinBUGS from R. *Journal of Statistical Software*, 12, 1–16.

**TABLES**

**Table S1.1. Satellite-tags used to track Kemp’s ridley (*Lepidochelys kempii*) and loggerhead (*Caretta caretta*) sea turtles in the Gulf of Mexico by site and year.** Grey areas indicate no tagging attempts at that site and year. A "." indicates 0 turtles tagged at that site/year. PAIS = Padre Island National Seashore, TX; RNMX = Rancho Nuevo, Tamalpais, Mexico; VCMX = Veracruz, Mexico; GS = Gulf Shores, Alabama; DRTO = Dry Tortugas National Park, Florida; SJP = St. Joseph Peninsula, Florida; EAFB = Eglin Air Force Base, Florida.

| **Location** | **Tag** | **1998** | **2000** | **2004** | **2005** | **2006** | **2007** | **2008** | **2009** | **2010** | **2011** | **2012** | **2013** | **Total** |
| --- | --- | --- | --- | --- | --- | --- | --- | --- | --- | --- | --- | --- | --- | --- |
| **GS** | SPOT 5 |  |  |  |  |  |  |  |  |  | 4 | 3 | 4 | **11** |
|  | SPLASH10 |  |  |  |  |  |  |  |  |  | 6 | 5 | 9 | **20** |
| **SJP** | SPOT 5 |  |  |  |  |  |  |  |  | 4 |  | 3 | 5 | **12** |
|  | SPLASH10 |  |  |  |  |  |  |  |  | . |  | 3 | 1 | **4** |
| **EAFB** | SPOT 5 |  |  |  |  |  |  |  |  |  |  | 2 | . | **2** |
| **DRTO** | SPOT 5 |  |  |  |  |  |  | 2 | 1 | 1 | 2 | . | 4 | **10** |
|  | SPLASH10 |  |  |  |  |  |  | . | 1 | . | 1 | 3 | . | **5** |
| **PAIS** | ST-6 | 1 | 1 | . | . | . | . | . |  | . | . | . | . | **2** |
|  | ST-20 | . | . | 2 | 1 | 3 | 1 | . |  | . | . | . | . | **7** |
|  | KS101 | . | . | . | . | . | . | 1 |  | 4 | 5 | 4 | 5 | **19** |
|  | MK-10AF | . | . | . | . | . | . | . |  | . | 4 | 4 | 5 | **13** |
| **RNMX** | KS101 |  |  |  |  |  |  |  |  | . | 4 |  |  | **4** |
|  | MK10A |  |  |  |  |  |  |  |  | . | 5 |  |  | **5** |
|  | MK10AF |  |  |  |  |  |  |  |  | 1 | . |  |  | **1** |
| **VCMX** | KS101 |  |  |  |  |  |  |  |  |  |  | 2 | . | **2** |
|  | SPOT5 |  |  |  |  |  |  |  |  |  |  | . | 10 | **10** |
|  | *Total* | **1** | **1** | **2** | **1** | **3** | **1** | **3** | **2** | **10** | **31** | **29** | **43** | 127 |

**Table S1.2. Sources of Kernel density estimates (KDEs) results, whether unpublished, previously published or summarized for a non-published report**. As stated in Methods, KDE analyses generally match those reported in this paper; however, specific methods can be found either with the published paper or in the supplementary material. Appendix S1 describes any methods that differed in these summaries from current methods.

| **Methods** | **Tag** | Methods |
| --- | --- | --- |
| 1 | 7689, 21811A, 47519, 47524, 47562, 62943, 70700, 82215, 100394, 100399, 100402, 100403, 100404, 101136, 106347 | Shaver et al. 2013 |
| 2 | 106343 | unpublished summary, 2013* |
| 3 | 101137, 101138, 101139, 112758, 112759, 112760, 112761, 112763, 112764, 112765, 112766, 112767, 117517 | unpublished summary, 2014* |
| 4 | 47755, 52968, 106360, 106361, 108170, 108172, 108173, 108174, 108964, 108965, 119923, 119924, 119938, 119941, 119942, 119943, 119944, 120438, 120439, 129497, 129498, 129499, 129500, 129502, 129504, 129505, 129506, 129507, 129508, 129510, 129511, 129512, 129513, 129514, 129515 | Hart, Lamont, Sartain, & Fujisaki, 2014 |
| 5 | 57656, 84715, 84716, 89971, 95898, 129490, 129493, 129495 | Hart, Lamont, Fujisaki, Tucker, & Carthy, 2012 |
| 6 | 47690, 47751, 47789, 47790, 62823, 91387, 101135, 106615, 106619, 119122, 119933, 119937, 126229, 126231, 126234, 126237 | this paper |

**Table S1.3. Foraging dates** **of Kemp’s ridley (*Lepidochelys kempii*) and loggerhead (*Caretta caretta*) sea turtles in the Gulf of Mexico.** Foraging dates determined by switching state-space modeling. Filtered locations were not always available for every foraging day.

| **Tag** | | **Start Foraging Period** | **End Foraging Period** | | **Days** | | **Filtered locations** |
| --- | --- | --- | --- | --- | --- | --- | --- |
| ***Kemp's ridleys*** | | |  | |  | |  |
| 7689 | | 7/12/1998 | 12/8/1998 | | 150 | | 40 |
| 21811A | | 4/24/2000 | 6/28/2000 | | 66 | | 143 |
|  | | 7/29/2000 | 9/26/2000 | | 60 | |  |
| 47789 | | 6/2/2004 | 6/6/2004 | | 5 | | 311 |
|  | | 7/4/2004 | 9/9/2004 | | 68 | |  |
|  | | 9/18/2004 | 12/21/2004 | | 95 | |  |
|  | | 12/28/2004 | 3/29/2005 | | 92 | |  |
| 47790 | | 6/28/2004 | 9/12/2004 | | 77 | | 87 |
|  | | 9/21/2004 | 9/25/2004 | | 5 | |  |
| 53631 | | 7/5/2005 | 9/23/2005 | | 81 | | 152 |
| 62822 | | 5/11/2006 | 5/11/2006 | | 1 | | 515 |
|  | | 6/15/2006 | 6/20/2006 | | 6 | |  |
|  | | 7/22/2006 | 4/4/2007 | | 257 | |  |
| 62943 | | 6/7/2006 | 12/31/2006 | | 208 | | 412 |
|  | | 1/7/2007 | 3/25/2007 | | 78 | |  |
| 62823 | | 6/18/2006 | 8/3/2006 | | 47 | | 60 |
| 70700 | | 7/9/2007 | 10/3/2007 | | 87 | | 95 |
| 82215 | | 6/20/2008 | 6/26/2008 | | 7 | | 204 |
|  | | 7/7/2008 | 9/2/2008 | | 58 | |  |
| 47519 | | 5/25/2010 | 5/25/2010 | | 1 | | 178 |
|  | | 6/8/2010 | 7/9/2010 | | 32 | |  |
|  | | 7/15/2010 | 8/28/2010 | | 45 | |  |
| 47524 | | 9/9/2010 | 10/18/2010 | | 40 | | 80 |
| 47562 | | 6/16/2010 | 7/4/2010 | | 19 | | 248 |
|  | | 7/14/2010 | 10/6/2010 | | 85 | |  |
| 47690 | | 6/6/2010 | 8/6/2010 | | 62 | | 66 |
| 101136 | | 4/27/2011 | 5/19/2011 | | 23 | | 465 |
|  | | 6/6/2011 | 9/10/2011 | | 97 | |  |
|  | | 9/14/2011 | 10/7/2011 | | 24 | |  |
| 101140 | | 6/3/2011 | 7/30/2011 | | 58 | | 259 |
| 106341 | | 7/2/2011 | 7/14/2011 | | 13 | | 208 |
|  | | 7/20/2011 | 9/7/2011 | | 50 | |  |
| 106343 | | 6/11/2011 | 6/12/2011 | | 2 | | 1066 |
|  | | 8/4/2011 | 7/31/2012 | | 363 | |  |
| 106346 | | 6/25/2011 | 9/16/2011 | | 84 | | 293 |
| 106347 | | 5/7/2011 | 5/26/2011 | | 20 | | 377 |
|  | | 6/4/2011 | 6/6/2011 | | 3 | |  |
|  | | 6/28/2011 | 6/28/2011 | | 1 | |  |
|  | | 7/6/2011 | 9/29/2011 | | 86 | |  |
| 100399 | | 9/20/2010 | 6/9/2011 | | 263 | | 374 |
| 100391 | | 9/13/2011 | 9/14/2011 | | 2 | | 1 |
| 100392 | | 6/19/2011 | 6/30/2011 | | 12 | | 420 |
|  | | 7/5/2011 | 7/20/2011 | | 16 | |  |
|  | | 7/26/2011 | 8/1/2011 | | 7 | |  |
|  | | 8/8/2011 | 12/6/2011 | | 121 | |  |
| 100393 | | 7/21/2011 | 10/28/2011 | | 100 | | 417 |
|  | | 11/3/2011 | 12/6/2011 | | 34 | |  |
| 100394 | | 8/6/2011 | 11/9/2011 | | 96 | | 251 |
| 100395 | | 7/12/2011 | 7/15/2011 | | 4 | | 412 |
|  | | 7/21/2011 | 8/13/2011 | | 24 | |  |
|  | | 8/17/2011 | 9/1/2011 | | 16 | |  |
|  | | 9/9/2011 | 12/7/2011 | | 90 | |  |
| 100402 | | 8/3/2011 | 9/17/2011 | | 46 | | 243 |
| 100403 | | 7/18/2011 | 7/18/2011 | | 1 | | 438 |
|  | | 7/31/2011 | 8/30/2011 | | 31 | |  |
|  | | 9/2/2011 | 12/7/2011 | | 97 | |  |
| 100404 | | 7/4/2011 | 7/6/2011 | | 3 | | 406 |
|  | | 7/13/2011 | 8/2/2011 | | 21 | |  |
|  | | 8/13/2011 | 8/16/2011 | | 4 | |  |
|  | | 8/20/2011 | 9/7/2011 | | 19 | |  |
|  | | 9/11/2011 | 12/7/2011 | | 88 | |  |
| 100406 | | 6/22/2011 | 6/25/2011 | | 4 | | 331 |
|  | | 7/5/2011 | 9/17/2011 | | 75 | |  |
| 117512 | | 5/29/2012 | 6/9/2012 | | 12 | | 44 |
|  | | 9/3/2012 | 9/18/2012 | | 16 | |  |
| 117513 | | 6/12/2012 | 7/30/2012 | | 49 | | 144 |
| 117514 | | 6/1/2012 | 7/18/2012 | | 48 | | 171 |
| 117516 | | 7/9/2012 | 9/22/2012 | | 76 | | 151 |
| 101137 | | 5/11/2011 | 5/25/2011 | | 15 | | 2508 |
|  | | 5/27/2011 | 8/13/2011 | | 79 | |  |
|  | | 8/16/2011 | 6/21/2012 | | 311 | |  |
|  | | 6/23/2012 | 8/27/2012 | | 66 | |  |
|  | | 8/30/2012 | 3/20/2013 | | 203 | |  |
|  | | 3/25/2013 | 9/12/2013 | | 172 | |  |
|  | | 9/17/2013 | 10/3/2013 | | 17 | |  |
|  | | 10/5/2013 | 10/21/2013 | | 17 | |  |
| 101138 | | 5/2/2011 | 5/7/2011 | | 6 | | 1732 |
|  | | 5/15/2011 | 5/16/2011 | | 2 | |  |
|  | | 7/2/2011 | 7/3/2011 | | 2 | |  |
|  | | 7/6/2011 | 10/19/2011 | | 106 | |  |
|  | | 10/26/2011 | 11/18/2011 | | 24 | |  |
|  | | 11/25/2011 | 12/1/2011 | | 7 | |  |
|  | | 12/4/2011 | 2/4/2012 | | 63 | |  |
|  | | 2/11/2012 | 2/18/2012 | | 8 | |  |
|  | | 3/4/2012 | 6/16/2012 | | 105 | |  |
|  | | 6/21/2012 | 8/30/2012 | | 71 | |  |
|  | | 9/3/2012 | 10/30/2012 | | 58 | |  |
|  | | 11/4/2012 | 11/9/2012 | | 6 | |  |
|  | | 11/16/2012 | 3/30/2013 | | 135 | |  |
| 101139 | | 6/26/2011 | 6/29/2011 | | 4 | | 1858 |
|  | | 7/19/2011 | 8/12/2011 | | 25 | |  |
|  | | 8/16/2011 | 9/2/2011 | | 18 | |  |
|  | | 9/7/2011 | 12/16/2011 | | 101 | |  |
|  | | 12/27/2011 | 5/4/2012 | | 130 | |  |
|  | | 5/6/2012 | 5/19/2012 | | 14 | |  |
|  | | 5/21/2012 | 5/23/2012 | | 3 | |  |
|  | | 5/31/2012 | 6/21/2012 | | 22 | |  |
|  | | 6/26/2012 | 7/2/2012 | | 7 | |  |
|  | | 7/10/2012 | 4/3/2013 | | 268 | |  |
|  | | 4/5/2013 | 5/12/2013 | | 38 | |  |
|  | | 5/14/2013 | 7/1/2013 | | 49 | |  |
|  | | 7/6/2013 | 7/10/2013 | | 5 | |  |
|  | | 7/12/2013 | 7/21/2013 | | 10 | |  |
|  | | 7/29/2013 | 8/6/2013 | | 9 | |  |
|  | | 8/8/2013 | 8/23/2013 | | 16 | |  |
|  | | 9/7/2013 | 9/11/2013 | | 5 | |  |
| 112758 | | 6/20/2012 | 6/21/2012 | | 2 | | 1949 |
|  | | 8/8/2012 | 11/21/2012 | | 106 | |  |
|  | | 11/24/2012 | 12/13/2012 | | 20 | |  |
|  | | 12/16/2012 | 10/20/2013 | | 309 | |  |
| 112759 | | 4/15/2012 | 4/18/2012 | | 4 | | 1189 |
|  | | 6/29/2012 | 7/4/2013 | | 371 | |  |
| 112760 | | 4/16/2012 | 4/17/2012 | | 2 | | 1774 |
|  | | 4/25/2012 | 4/27/2012 | | 3 | |  |
|  | | 5/4/2012 | 5/4/2012 | | 1 | |  |
|  | | 5/11/2012 | 5/13/2012 | | 3 | |  |
|  | | 5/19/2012 | 5/23/2012 | | 5 | |  |
|  | | 5/26/2012 | 7/29/2012 | | 65 | |  |
|  | | 8/2/2012 | 11/10/2012 | | 101 | |  |
|  | | 11/16/2012 | 2/6/2013 | | 83 | |  |
|  | | 2/8/2013 | 2/23/2013 | | 16 | |  |
|  | | 2/28/2013 | 3/1/2013 | | 2 | |  |
|  | | 3/7/2013 | 4/25/2013 | | 50 | |  |
|  | | 5/4/2013 | 5/4/2013 | | 1 | |  |
|  | | 5/6/2013 | 5/9/2013 | | 4 | |  |
|  | | 5/13/2013 | 5/25/2013 | | 13 | |  |
|  | | 6/5/2013 | 6/6/2013 | | 2 | |  |
|  | | 6/19/2013 | 6/20/2013 | | 2 | |  |
|  | | 6/23/2013 | 6/29/2013 | | 7 | |  |
|  | | 7/15/2013 | 10/20/2013 | | 98 | |  |
| 112761 | | 5/18/2012 | 5/21/2012 | | 4 | | 1062 |
|  | | 6/13/2012 | 6/21/2012 | | 9 | |  |
|  | | 7/14/2012 | 7/19/2012 | | 6 | |  |
|  | | 7/28/2012 | 8/28/2012 | | 32 | |  |
|  | | 9/17/2012 | 9/26/2012 | | 10 | |  |
|  | | 10/2/2012 | 4/7/2013 | | 188 | |  |
|  | | 5/1/2013 | 5/11/2013 | | 11 | |  |
|  | | 5/27/2013 | 5/30/2013 | | 4 | |  |
|  | | 6/7/2013 | 7/8/2013 | | 32 | |  |
|  | | 7/12/2013 | 7/27/2013 | | 16 | |  |
|  | | 7/30/2013 | 8/10/2013 | | 12 | |  |
|  | | 8/15/2013 | 8/23/2013 | | 9 | |  |
|  | | 9/20/2013 | 10/16/2013 | | 27 | |  |
|  | | 10/19/2013 | 10/21/2013 | | 3 | |  |
| 112763 | | 6/25/2013 | 7/7/2013 | | 13 | | 285 |
|  | | 7/10/2013 | 8/10/2013 | | 32 | |  |
|  | | 8/23/2013 | 9/3/2013 | | 12 | |  |
|  | | 9/10/2013 | 9/13/2013 | | 4 | |  |
|  | | 10/9/2013 | 10/20/2013 | | 12 | |  |
| 112764 | | 5/11/2013 | 5/11/2013 | | 1 | | 117 |
|  | | 5/13/2013 | 5/15/2013 | | 3 | |  |
|  | | 5/29/2013 | 6/11/2013 | | 14 | |  |
|  | | 7/9/2013 | 7/9/2013 | | 1 | |  |
|  | | 7/31/2013 | 9/5/2013 | | 37 | |  |
|  | | 9/11/2013 | 9/12/2013 | | 2 | |  |
| 112765 | | 5/18/2013 | 6/22/2013 | | 36 | | 672 |
|  | | 6/25/2013 | 7/22/2013 | | 28 | |  |
|  | | 7/25/2013 | 10/20/2013 | | 88 | |  |
| 112766 | | 6/6/2013 | 6/11/2013 | | 6 | | 457 |
|  | | 6/13/2013 | 7/23/2013 | | 41 | |  |
|  | | 7/25/2013 | 8/7/2013 | | 14 | |  |
|  | | 8/9/2013 | 8/14/2013 | | 6 | |  |
|  | | 8/16/2013 | 9/4/2013 | | 20 | |  |
|  | | 9/6/2013 | 10/7/2013 | | 32 | |  |
|  | | 10/11/2013 | 10/21/2013 | | 11 | |  |
| 112767 | | 6/12/2013 | 6/26/2013 | | 15 | | 296 |
|  | | 7/19/2013 | 8/21/2013 | | 34 | |  |
|  | | 9/21/2013 | 10/21/2013 | | 31 | |  |
| 117517 | | 7/12/2013 | 7/27/2013 | | 16 | | 219 |
|  | | 7/30/2013 | 8/20/2013 | | 22 | |  |
| 117518 | | 6/17/2013 | 9/22/2013 | | 98 | | 800 |
| 117519 | | 6/24/2013 | 7/22/2013 | | 29 | | 439 |
|  | | 7/29/2013 | 8/9/2013 | | 12 | |  |
| 117520 | | 6/19/2013 | 7/17/2013 | | 29 | | 340 |
| 117521 | | 7/25/2013 | 9/1/2013 | | 39 | | 240 |
| 101134 | | 6/16/2012 | 6/19/2012 | | 4 | | 7 |
| 126229 | | 5/11/2013 | 5/14/2013 | | 4 | | 138 |
|  | | 5/29/2013 | 7/17/2013 | | 50 | |  |
| 126230 | | 6/23/2013 | 8/12/2013 | | 51 | | 174 |
| 126231 | | 5/20/2013 | 7/8/2013 | | 50 | | 130 |
| 126232 | | 7/4/2013 | 7/21/2013 | | 18 | | 52 |
| 1011351 (before gap) | | 8/5/2012 | 10/2/2013 | | 424 | | 1617 |
| 1011352 (after gap) | | 11/10/2013 | 2/26/2014 | | 109 | | 186 |
| 126228 | | 7/9/2013 | 7/19/2013 | | 11 | | 34 |
| 1262331 (Before gap) | | none |  | |  | | 0 |
| 1262332 (After gap) | | 2/10/2014 | 4/1/2014 | | 51 | | 4 |
| 126234 | | 6/25/2013 | 9/16/2013 | | 84 | | 241 |
| 126235 | | 6/21/2013 | 7/28/2013 | | 38 | | 76 |
| 126236 | | 7/13/2013 | 7/20/2013 | | 8 | | 0 |
| 126237 | | 8/14/2013 | 12/6/2013 | | 115 | | 282 |
| 119937 | | 6/13/2012 | 8/31/2012 | | 80 | | 398 |
| **Tag** | | **Start Foraging Period** | **End Foraging Period** | | **Days** | | **Filtered locations** |
| ***Loggerheads*** | | |  |  | |  | |
| 53000 | 7/28/2012 | | 8/27/2012 | 31 | | 144 | |
| 106337 | 6/24/2011 | | 7/12/2011 | 19 | | 106 | |
|  | 7/15/2011 | | 8/7/2011 | 24 | |  | |
|  | 8/14/2011 | | 8/31/2011 | 18 | |  | |
| 53017 | 6/4/2012 | | 6/26/2012 | 23 | | 92 | |
| 119952a | 6/29/2012 | | 7/6/2012 | 8 | | 24 | |
| 108170 | 7/25/2011 | | 3/1/2012 | 221 | | 492 | |
| 106360 | 8/31/2011 | | 12/24/2011 | 116 | | 196 | |
| 108172 | 8/10/2011 | | 2/25/2012 | 200 | | 448 | |
| 108173 | 7/19/2011 | | 4/2/2012 | 259 | | 659 | |
| 108174 | 8/16/2011 | | 11/7/2011 | 84 | | 143 | |
| 106361 | 7/1/2011 | | 6/18/2012 | 354 | | 745 | |
| 108961 | 7/31/2011 | | 8/17/2011 | 18 | | 233 | |
| 108964 | 8/21/2011 | | 9/14/2011 | 25 | | 149 | |
| 108965 | 8/11/2011 | | 7/21/2012 | 346 | | 1166 | |
| 119943 | 8/2/2012 | | 11/20/2012 | 111 | | 274 | |
| 119924 | 7/16/2012 | | 9/10/2012 | 57 | | 426 | |
| 119946 | 7/17/2012 | | 9/4/2012 | 50 | | 243 | |
| 119942 | 8/5/2012 | | 9/1/2012 | 28 | | 296 | |
|  | 9/4/2012 | | 9/29/2012 | 26 | |  | |
| 119948 | 8/12/2012 | | 10/12/2012 | 62 | | 197 | |
| 119952 | 8/13/2012 | | 10/3/2012 | 52 | | 124 | |
| 120438 | 8/24/2012 | | 10/13/2013 | 416 | | 1483 | |
| 129496 | 8/22/2013 | | 9/5/2013 | 15 | | 155 | |
|  | 9/13/2013 | | 9/19/2013 | 7 | |  | |
|  | 9/28/2013 | | 10/15/2013 | 18 | |  | |
| 129497 | 7/21/2013 | | 10/12/2013 | 84 | | 540 | |
| 129498 | 7/3/2013 | | 10/15/2013 | 105 | | 547 | |
| 129499 | 9/2/2013 | | 10/15/2013 | 44 | | 296 | |
| 129500 | 8/16/2013 | | 10/15/2013 | 61 | | 328 | |
| 129502 | 8/6/2013 | | 10/15/2013 | 71 | | 941 | |
| 129503 | 8/5/2013 | | 10/15/2013 | 72 | | 650 | |
| 129504 | 7/23/2013 | | 10/14/2013 | 84 | | 507 | |
| 129505 | 8/20/2013 | | 10/15/2013 | 57 | | 815 | |
| 129506 | 8/5/2013 | | 8/27/2013 | 23 | | 339 | |
|  | 9/10/2013 | | 10/15/2013 | 36 | |  | |
| 129507 | 9/6/2013 | | 10/15/2013 | 40 | | 707 | |
| 129508 | 8/18/2013 | | 10/15/2013 | 59 | | 445 | |
| 129509 | 8/4/2013 | | 9/18/2013 | 46 | | 653 | |
|  | 9/21/2013 | | 10/15/2013 | 25 | |  | |
| 129510 | 8/7/2013 | | 10/14/2013 | 69 | | 419 | |
| 129511 | 8/8/2013 | | 9/23/2013 | 47 | | 270 | |
| 129512 | 7/26/2013 | | 10/15/2013 | 82 | | 670 | |
| 129513 | 7/22/2013 | | 8/12/2013 | 22 | | 201 | |
| 129514 | 7/31/2013 | | 10/15/2013 | 77 | | 701 | |
| 129515 | 8/9/2013 | | 9/22/2013 | 45 | | 298 | |
| 119941 | 9/22/2012 | | 9/23/2012 | 2 | | 360 | |
|  | 9/26/2012 | | 8/31/2013 | 340 | |  | |
| 119938 | 8/7/2012 | | 2/22/2013 | 200 | | 529 | |
| 119944 | 6/28/2012 | | 7/15/2013 | 383 | | 774 | |
| 119923 | 7/31/2012 | | 1/7/2013 | 161 | | 741 | |
| 120439 | 8/31/2012 | | 1/9/2013 | 132 | | 649 | |
|  | 1/14/2013 | | 4/6/2013 | 83 | |  | |
|  | 4/18/2013 | | 4/23/2013 | 6 | |  | |
|  | 5/10/2013 | | 5/24/2013 | 15 | |  | |
| 52968 | 8/21/2010 | | 9/29/2011 | 405 | | 1067 | |
|  | 10/3/2011 | | 3/6/2012 | 156 | |  | |
| 47755 | 9/7/2010 | | 1/4/2011 | 120 | | 325 | |
| 57656 | 8/16/2010 | | 9/12/2010 | 28 | | 49 | |
| 89971 | 8/24/2010 | | 9/15/2010 | 23 | | 128 | |
| 84715 | 7/13/2008 | | 9/11/2008 | 61 | | 88 | |
| 84716 | 5/25/2008 | | 7/16/2008 | 53 | | 235 | |
|  | 7/22/2008 | | 8/23/2008 | 33 | |  | |
| 95898 | 7/23/2009 | | 9/19/2009 | 59 | | 333 | |
|  | 9/23/2009 | | 10/26/2009 | 34 | |  | |
| 129488 | 8/26/2013 | | 9/25/2013 | 31 | | 110 | |
| 129490 | 7/30/2013 | | 1/22/2014 | 177 | | 597 | |
| 129493 | 7/17/2013 | | 11/30/2013 | 137 | | 892 | |
|  | 12/5/2013 | | 12/7/2013 | 3 | |  | |
| 129495 | 8/15/2013 | | 11/19/2013 | 97 | | 1026 | |
| 47751 | 7/30/2010 | | 9/16/2012 | 780 | | 1785 | |
| 106615 | 7/1/2011 | | 1/7/2012 | 191 | | 411 | |
| 106618 | 7/5/2011 | | 11/27/2011 | 146 | | 5 | |
|  | 12/11/2011 | | 1/3/2012 | 24 | |  | |
| 106619 | 6/20/2011 | | 7/4/2011 | 15 | | 118 | |
|  | 7/18/2011 | | 7/20/2011 | 3 | |  | |
|  | 7/24/2011 | | 7/26/2011 | 3 | |  | |
|  | 8/1/2011 | | 12/15/2011 | 137 | |  | |
| 119122 | 7/30/2012 | | 10/12/2012 | 75 | | 669 | |
| 119127 | 7/4/2012 | | 11/30/2012 | 150 | | 21 | |
| 119933 | 8/3/2012 | | 8/1/2013 | 364 | | 1913 | |
| 91387 | 6/20/2009 | | 6/30/2009 | 11 | | 207 | |
|  | 7/6/2009 | | 1/17/2010 | 196 | |  | |

**Table S1.4.** **Home range overlap (95% Kernel Density Estimates [KDE]) with dominant (d) and subdominant (sd) sediment types for Kemp’s ridley (*Lepidochelys kempii*) and loggerhead (*Caretta caretta*) sea turtles in the Gulf of Mexico.** Sediments were classified as mud, sand, gravel (grav) and rock; data from the Dominant Sediments layer from the Gulf of Mexico Data Atlas (https://www.ncddc.noaa.gov/; accessed 20 February 2018). Values are the number of grid cells classified as “d” or “sd” for each sediment. The overall dominant type (Dom Type) for each KDE was classified by the maximum grid cell representation (bold). The percent coverage indicates how much of the 95% KDE had sediment grid cells within it. For turtles with more than one KDE, separate KDEs are denoted with letters. The first line represents areas where 95% KDEs from Kemp's ridleys and loggerheads intersected.

| **95% KDE** | **Total cells** | **Mud, sd** | **Mud, d** | **Sand, sd** | | | **Sand, d** | **Grav, sd** | | | | **Grav, d** | | **Rock, sd** | **Rock, d** | | **Dom Type** | **Percent coverage** | | | |
| --- | --- | --- | --- | --- | --- | --- | --- | --- | --- | --- | --- | --- | --- | --- | --- | --- | --- | --- | --- | --- | --- |
| Intersect | 947 | 148 | 160 | 192 | | | **374** | 35 | | | | 33 | | 4 | 0 | | sand, d | 96 | | | |
| ***Kemp's ridleys*** | |  | | | | | | | | | | | | | | |  | | | | |
| 7689 | 899 | 4 | 2 | **514** | | | 272 | 96 | | | | 11 | | 0 | 0 | | sand, sd mud, sd sand, d mud, d mud, d mud, d mud, d mud, d mud, d sand, d mud, sd sand, d mud, d mud, d sand, d mud, sd sand, sd mud, d mud, d mud, d mud, d mud, d sand, sd mud, d mud, d mud, d mud, d mud, d mud, sd mud, d mud, d mud, d mud, d mud, d mud, d mud, d mud, d mud, d sand, d sand, d mud, sd sand, d | 94  100  100  99  100  95  97  98  100  94  100  99  100  99  100  94  69  100  100  91  100  97  92  100  99  94  100  100  99  100  100  99  98  98  94  99  100  99  91  99  100  100 | | | |
| 47519 | 669 | **242** | 151 | 151 | | | 100 | 22 | | | | 3 | | 0 | 0 | |  |  |  |  |  |
| 47524 | 62 | 3 | 3 | 7 | | | **47** | 0 | | | | 0 | | 2 | 0 | |  |  |  |  |  |
| 47562 | 566 | 61 | **478** | 12 | | | 12 | 1 | | | | 2 | | 0 | 0 | |  |  |  |  |  |
| 47562 | 646 | 99 | **410** | 49 | | | 88 | 0 | | | | 0 | | 0 | 0 | |  |  |  |  |  |
| 47690 | 3686 | 977 | **1896** | 375 | | | 399 | 33 | | | | 6 | | 0 | 0 | |  |  |  |  |  |
| 47790 | 757 | 203 | **230** | 110 | | | 181 | 14 | | | | 19 | | 0 | 0 | |  |  |  |  |  |
| 62823 | 1956 | 552 | **761** | 337 | | | 192 | 81 | | | | 32 | | 1 | 0 | |  |  |  |  |  |
| 62943 | 996 | 239 | **471** | 111 | | | 155 | 18 | | | | 2 | | 0 | 0 | |  |  |  |  |  |
| 70700 | 1124 | 202 | 290 | 222 | | | **376** | 21 | | | | 6 | | 5 | 2 | |  |  |  |  |  |
| 82215 | 578 | **192** | 126 | 129 | | | 106 | 22 | | | | 3 | | 0 | 0 | |  |  |  |  |  |
| 100394 | 208 | 9 | 12 | 50 | | | **133** | 2 | | | | 0 | | 2 | 0 | |  |  |  |  |  |
| 100403 | 678 | 141 | **370** | 61 | | | 86 | 11 | | | | 9 | | 0 | 0 | |  |  |  |  |  |
| 100404 | 455 | 19 | **427** | 3 | | | 3 | 0 | | | | 3 | | 0 | 0 | |  |  |  |  |  |
| 106343 | 182 | 0 | 0 | 0 | | | **167** | 0 | | | | 0 | | 11 | 4 | |  |  |  |  |  |
| 106347 | 324 | **110** | 93 | 53 | | | 44 | 11 | | | | 13 | | 0 | 0 | |  |  |  |  |  |
| 112759 | 93 | 2 | 2 | **54** | | | 28 | 6 | | | | 0 | | 1 | 0 | |  |  |  |  |  |
| 112761 | 167 | 18 | **143** | 6 | | | 0 | 0 | | | | 0 | | 0 | 0 | |  |  |  |  |  |
| 112763 | 444 | 80 | **219** | 62 | | | 64 | 18 | | | | 1 | | 0 | 0 | |  |  |  |  |  |
| 112764 | 224 | 11 | **195** | 11 | | | 3 | 4 | | | | 0 | | 0 | 0 | |  |  |  |  |  |
| 112767 | 873 | 230 | **349** | 135 | | | 134 | 22 | | | | 3 | | 0 | 0 | |  |  |  |  |  |
| 117517 | 1108 | 221 | **623** | 105 | | | 131 | 25 | | | | 3 | | 0 | 0 | |  |  |  |  |  |
| 119937 | 96 | 0 | 0 | **39** | | | 26 | 26 | | | | 5 | | 0 | 0 | |  |  |  |  |  |
| 126229 | 3169 | 720 | **1684** | 335 | | | 378 | 42 | | | | 10 | | 0 | 0 | |  |  |  |  |  |
| 126231 | 2276 | 785 | **1142** | 250 | | | 91 | 8 | | | | 0 | | 0 | 0 | |  |  |  |  |  |
| 100404a | 1116 | 85 | **978** | 26 | | | 19 | 5 | | | | 3 | | 0 | 0 | |  |  |  |  |  |
| 100404b | 762 | 107 | **436** | 69 | | | 144 | 6 | | | | 0 | | 0 | 0 | |  |  |  |  |  |
| 101136a | 1259 | 260 | **788** | 121 | | | 70 | 17 | | | | 3 | | 0 | 0 | |  |  |  |  |  |
| 101136b | 1501 | **419** | 393 | 353 | | | 270 | 59 | | | | 7 | | 0 | 0 | |  |  |  |  |  |
| 101137a | 3117 | 829 | **1095** | 452 | | | 654 | 73 | | | | 14 | | 0 | 0 | |  |  |  |  |  |
| 101137b | 3231 | 899 | **1644** | 270 | | | 355 | 46 | | | | 6 | | 11 | 0 | |  |  |  |  |  |
| 101138a | 189 | 51 | **72** | 23 | | | 40 | 1 | | | | 2 | | 0 | 0 | |  |  |  |  |  |
| 101138b | 141 | 30 | **61** | 17 | | | 28 | 1 | | | | 4 | | 0 | 0 | |  |  |  |  |  |
| 101138c | 813 | 189 | **447** | 82 | | | 80 | 10 | | | | 5 | | 0 | 0 | |  |  |  |  |  |
| 101138d | 513 | 134 | **199** | 69 | | | 95 | 8 | | | | 8 | | 0 | 0 | |  |  |  |  |  |
| 101139a | 491 | 76 | **350** | 42 | | | 13 | 7 | | | | 3 | | 0 | 0 | |  |  |  |  |  |
| 101139b | 806 | 69 | **700** | 15 | | | 11 | 3 | | | | 4 | | 1 | 3 | |  |  |  |  |  |
| 101139c | 451 | 65 | **277** | 51 | | | 43 | 11 | | | | 4 | | 0 | 0 | |  |  |  |  |  |
| 112758a | 308 | 9 | 50 | 30 | | | **212** | 1 | | | | 0 | | 5 | 1 | |  |  |  |  |  |
| 112758b | 327 | 26 | 66 | 31 | | | **149** | 11 | | | | 35 | | 9 | 0 | |  |  |  |  |  |
| 112760a | 1659 | **565** | 552 | 319 | | | 170 | 40 | | | | 13 | | 0 | 0 | |  |  |  |  |  |
| 112760b | 85 | 0 | 0 | 23 | | | **62** | 0 | | | | 0 | | 0 | 0 | |  |  |  |  |  |
| 112760c | 114 | 38 | **69** | | 5 | 2 | | | 0 | | | | 0 | 0 | 0 | | mud, d | | | 100 | |
| 112765a | 1191 | 118 | 372 | | 218 | **471** | | | 10 | | | | 2 | 0 | 0 | | sand, d | | | 74 | |
| 112765b | 916 | 45 | **510** | | 91 | 270 | | | 0 | | | | 0 | 0 | 0 | | mud, d | | | 91 | |
| 112766a | 5005 | 1218 | **1893** | | 790 | 991 | | | 98 | | | | 15 | 0 | 0 | | mud, d | | | 99 | |
| 112766b | 3994 | 494 | **1640** | | 620 | 1053 | | | 106 | | | | 68 | 11 | 1 | | mud, d | | | 93 | |
| 21811a | 1771 | 341 | **1140** | | 166 | 87 | | | 26 | | | | 7 | 3 | 1 | | mud, d | | | 100 | |
| 21811b | 4733 | **1793** | 1516 | | 710 | 575 | | | 89 | | | | 34 | 15 | 1 | | mud, sd | | | 99 | |
| 47789a | 1272 | 157 | **645** | | 103 | 361 | | | 6 | | | | 0 | 0 | 0 | | mud, d | | | 100 | |
| 47789b | 2042 | 379 | **1063** | | 234 | 336 | | | 27 | | | | 3 | 0 | 0 | | mud, d | | | 100 | |
| 47789c | 1638 | 343 | **811** | | 186 | 264 | | | 21 | | | | 1 | 11 | 1 | | mud, d | | | 100 | |
| ***Loggerheads*** | | | | | | | | | | | | | | | | | | | | | |
| 47751 | 250 | 35 | 43 | | **73** | 52 | | | 18 | | | | 6 | 12 | 8 | | sand, sd | | | 97 | |
| 57656 | 229 | 21 | 0 | | **179** | 29 | | | 0 | | | | 0 | 0 | 0 | | sand, sd | | | 99 | |
| 84715 | 24 | 5 | 1 | | **7** | 6 | | | 5 | | | | 0 | 0 | 0 | | sand, sd | | | 81 | |
| 84716 | 45 | 3 | 0 | | **24** | 6 | | | 9 | | | | 0 | 2 | 1 | | sand, sd | | | 100 | |
| 89971 | 78 | 0 | 0 | | **64** | 0 | | | 14 | | | | 0 | 0 | 0 | | sand, sd | | | 100 | |
| 91387 | 112 | 18 | **45** | | 7 | 0 | | | 7 | | | | 22 | 13 | 0 | | mud, d | | | 97 | |
| 95898 | 123 | 0 | 0 | | 32 | **63** | | | 12 | | | | 7 | 1 | 8 | | sand, d | | | 100 | |
| 106360 | 216 | 0 | 0 | | 48 | 13 | | | **117** | | | | 29 | 2 | 7 | | grav, sd | | | 100 | |
| 106361 | 955 | 1 | 6 | | 153 | **656** | | | 39 | | | | 87 | 7 | 6 | | sand, d | | | 100 | |
| 106615 | 144 | 0 | 0 | | 45 | **92** | | | 5 | | | | 2 | 0 | 0 | | sand, d | | | 99 | |
| 106619 | 50 | 4 | **33** | | 5 | 2 | | | 0 | | | | 0 | 5 | 1 | | mud, d | | | 63 | |
| 108170 | 178 | 0 | 0 | | 43 | **109** | | | 16 | | | | 9 | 0 | 0 | | sand, d | | | 91 | |
| 108172 | 249 | 0 | 0 | | 73 | 52 | | | **95** | | | | 22 | 2 | 5 | | grav, sd | | | 100 | |
| 108173 | 134 | 0 | 0 | | **40** | 12 | | | 26 | | | | 7 | 32 | 17 | | sand, sd | | | 100 | |
| 108174 | 113 | 13 | 20 | | 8 | **71** | | | 0 | | | | 0 | 1 | 0 | | sand, d | | | 100 | |
| 108964 | 374 | 0 | 0 | | **141** | 93 | | | 92 | | | | 15 | 14 | 19 | | sand, sd | | | 100 | |
| 108965 | 276 | 8 | 0 | | 41 | **158** | | | 25 | | | | 44 | 0 | 0 | | sand, d | | | 97 | |
| 119122 | 16 | 0 | **9** | | 0 | 0 | | | 0 | | | | 0 | 3 | 4 | | mud, d | | | 81 | |
| 119923 | 44 | 3 | **24** | | 7 | 9 | | | 1 | | | | 0 | 0 | 0 | | mud, d | | | 100 | |
| 119924 | 191 | 0 | 0 | | 59 | **77** | | | 18 | | | | 25 | 6 | 6 | | sand, d | | | 100 | |
| 119938 | 79 | 0 | 0 | | 4 | **44** | | | 0 | | | | 0 | 22 | 9 | | sand, d | | | 100 | |
| 119941 | 48 | 2 | 15 | | 8 | **21** | | | 0 | | | | 0 | 2 | 0 | | sand, d | | | 94 | |
| 119942 | 279 | 88 | **133** | | 28 | 24 | | | 4 | | | | 2 | 0 | 0 | | mud, d | | | 97 | |
| 119943 | 69 | 3 | 0 | | 20 | **28** | | | 18 | | | | 0 | 0 | 0 | | sand, d | | | 100 | |
| 119944 | 119 | 0 | 6 | | 36 | **43** | | | 16 | | | | 18 | 0 | 0 | | sand, d | | | 100 | |
| 129490 | 17 | 2 | **13** | | 1 | 1 | | | 0 | | | | 0 | 0 | 0 | | mud, d | | | 94 | |
| 129493 | 32 | 0 | 0 | | 0 | **32** | | | 0 | | | | 0 | 0 | 0 | | sand, d | | | 100 | |
| 129497 | 21 | 0 | 0 | | 6 | **15** | | | 0 | | | | 0 | 0 | 0 | | sand, d | | | 100 | |
| 129498 | 44 | 1 | 2 | | 2 | **16** | | | 8 | | | | 15 | 0 | 0 | | sand, d | | | 86 | |
| 129500 | 136 | 0 | 0 | | **67** | 32 | | | 8 | | | | 0 | 12 | 17 | | sand, sd | | | 100 | |
| 129502 | 77 | 5 | **43** | | 11 | 18 | | | 0 | | | | 0 | 0 | 0 | | mud, d | | | 100 | |
| 129504 | 436 | 0 | 0 | | **185** | 104 | | | 117 | | | | 23 | 5 | 2 | | sand, sd | | | 100 | |
| 129505 | 23 | 0 | 0 | | 0 | **21** | | | 0 | | | | 0 | 0 | 0 | | sand, d | | | 78 | |
| 129506 | 253 | 0 | 0 | | 67 | 26 | | | **108** | | | | 52 | 0 | 0 | | grav, sd | | | 100 | |
| 129510 | 69 | 0 | 0 | | 3 | **59** | | | 0 | | | | 7 | 0 | 0 | | sand, d | | | 100 | |
| 129511 | 156 | 14 | 6 | | **56** | 48 | | | 9 | | | | 22 | 0 | 0 | | sand, sd | | | 100 | |
| 129512 | 72 | 0 | 0 | 4 | | **68** | | | | 0 | 0 | | | 0 | 0 | sand, d | | | 100 | |  |
| 129513 | 94 | **47** | 19 | 26 | | 2 | | | | 0 | 0 | | | 0 | 0 | mud, sd | | | 100 | |  |
| 129514 | 35 | 0 | 0 | **17** | | 13 | | | | 4 | 0 | | | 1 | 0 | sand, sd | | | 100 | |  |
| 129515 | 66 | 0 | 0 | 9 | | **27** | | | | 6 | 24 | | | 0 | 0 | sand, d | | | 100 | |  |
| 52968a | 437 | 4 | 0 | 49 | | **305** | | | | 8 | 2 | | | 54 | 15 | sand, d | | | 100 | |  |
| 52968b | 158 | 1 | 0 | 13 | | **89** | | | | 7 | 0 | | | 33 | 15 | sand, d | | | 100 | |  |

**Table S1.5.** **Summary table of the home range overlap with dominant (d) and subdominant (sd) sediment types for foraging home ranges for Kemp’s ridley (*Lepidochelys kempii*) and loggerhead (*Caretta caretta*) sea turtles in the Gulf of Mexico.** Sediments were classified as mud, sand, gravel (grav) and rock; data from the Dominant Sediments layer from the Gulf of Mexico Data Atlas (https://www.ncddc.noaa.gov/; accessed 20 February 2018). Values are the number of home ranges (95% Kernel Density Estimates) with the overall dominant habitat type (see Table S1.4 for individual breakdown).

|  | **Mud, d** | **Mud, sd** | **Sand, d** | **Sand, sd** | **Grav, d** | **Grav, sd** | **Rock, d** | **Rock, sd** |
| --- | --- | --- | --- | --- | --- | --- | --- | --- |
| **Kemp's ridley** | **35** | 6 | 8 | 3 | 0 | 0 | 0 | 0 |
| **Loggerheads** | 7 | 1 | **20** | 11 | 0 | 3 | 0 | 0 |
